# Cilia‐Mediated Insulin/Akt and ST2/JNK Signaling Pathways Regulate the Recovery of Muscle Injury

**DOI:** 10.1002/advs.202202632

**Published:** 2022-11-14

**Authors:** Daishi Yamakawa, Junya Tsuboi, Kousuke Kasahara, Chise Matsuda, Yuhei Nishimura, Tatsuya Kodama, Naoyuki Katayama, Masatoshi Watanabe, Masaki Inagaki

**Affiliations:** ^1^ Department of Physiology Mie University Graduate School of Medicine Tsu Mie 514‐8507 Japan; ^2^ Department of Gastroenterology and Hepatology Mie University Graduate School of Medicine Tsu Mie 514‐8507 Japan; ^3^ Department of Oncogenic Pathology Mie University Graduate School of Medicine Tsu Mie 514‐8507 Japan; ^4^ Department of Integrative Pharmacology Mie University Graduate School of Medicine Tsu Mie 514‐8507 Japan; ^5^ Department of Hematology and Oncology Mie University Graduate School of Medicine Tsu Mie 514‐8507 Japan

**Keywords:** adipogenesis, fibro/adipogenic progenitors, interleukin 13, interleukin 33, lipid raft, primary cilia, skeletal muscle regeneration

## Abstract

Following injury, skeletal muscle regenerates but fatty tissue accumulation is seen in aged muscle or muscular dystrophies. Fibro/adipogenic progenitors (FAPs) are key players in these events; however, the effect of primary cilia on FAPs remains unclear. Here, it is reported that genetic ablation of trichoplein (TCHP), a ciliary regulator, induces ciliary elongation on FAPs after injury, which promotes muscle regeneration while inhibiting adipogenesis. The defective adipogenic differentiation of FAPs is attributed to dysfunction of cilia‐dependent lipid raft dynamics, which is critical for insulin/Akt signaling. It is also found that interleukin (IL) 13 is substantially produced by intramuscular FAPs, which are upregulated by ciliary elongation and contribute to regeneration. Mechanistically, upon injury, long cilia excessively activate the IL33/ST2/JNK axis to enhance IL13 production, facilitating myoblast proliferation and M2 macrophage polarization. The results indicate that FAPs organize the regenerative responses to skeletal muscle injury via cilia‐mediated insulin/Akt and ST2/JNK signaling pathways.

## Graphical abstract

Skeletal muscle regeneration requires functional crosstalk between muscle cells and fibro/adipogenic progenitors (FAPs). In this article, it is shown that primary ciliary elongation on FAPs suppresses intramuscular adipogenesis after muscle injury by disturbing lipid raft‐dependent insulin/Akt signaling and also contributes to muscle regeneration by promoting myoblast proliferation and M2 macrophage infiltration via IL33/ST2/JNK pathway‐dependent IL13 production.

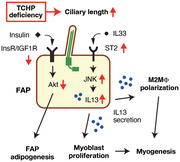

## 1 Introduction

Skeletal muscle is composed of multinucleated contractile myofibers, which are formed during development by the proliferative growth and fusion of mononucleated muscle cells. During post‐natal growth, the number of myofibers remains constant and skeletal muscle has a robust capacity to regenerate following injury. The potency of skeletal muscle regeneration depends primarily upon myogenic stem cells called satellite cells. Most satellite cells are mitotically quiescent under homeostatic conditions; however, upon skeletal muscle injury, they activate and enter the cell cycle, which yields daughter cells that self‐renew the pool of satellite cells or that differentiate into myoblasts to form new functional myofibers.^[^ ^1^, ^2^, ^3^ ^]^

The myogenic program requires functional cross‐talk between satellite cells/myoblasts and other resident cells in the skeletal muscle niche such as fibro/adipogenic progenitor cells (FAPs).^[^ ^4^, ^5^, ^6^ ^]^ FAPs are defined as multi‐potent progenitors that are able to differentiate into fibroblasts, adipocytes, and osteoclasts, but not into myoblasts.^[^ ^7^, ^8^ ^]^ Under quiescent conditions, FAPs reside in the stromal space between myofibers, but upon muscle injury, they become activated, proliferate, and secrete several cytokines, including interleukin (IL) 6, IL10, and follistatin, which promote myogenic differentiation.^[^ ^8^, ^9^, ^10^ ^]^ Furthermore, in skeletal muscle, FAPs are the main cellular source of IL33, a member of the IL1 family of cytokines.^[^ ^11^ ^]^ IL33 is a nuclear chromatin‐associated cytokine and is thought to be released from cells undergoing mechanical stress.^[^ ^12^ ^]^ The released IL33 triggers the recruitment of FOXP3^+^ regulatory T cells (Tregs) to injured muscle through the cell surface receptor ST2 (also known as IL1RL1). Tregs then secrete amphiregulin, a ligand for epidermal growth factor receptor (EGFR), to enhance regeneration.^[^ ^13^ ^]^ The crucial role of FAPs in muscle homeostasis and repair has also been established by studies showing a serious deficit of myofiber maintenance and regeneration in mice in which FAPs were genetically eliminated.^[^ ^14^, ^15^ ^]^

Ectopic deposition of excessive FAP‐derived adipocytes also occurs in skeletal muscle upon injury and various pathological conditions, including sarcopenia and some kinds of myopathy.^[^ ^16^, ^17^, ^18^ ^]^ A previous study revealed that the majority of FAPs dynamically form primary cilia, solitary nonmotile structures that project from the cell surface, during muscle regeneration. The Hedgehog (Hh) signal transduction pathway, which is essential for the development and patterning of numerous organ systems in vertebrates, depends on cilia, and Hh pathway components are enriched in cilia. When cilia are genetically removed from FAPs, the expression of Hh target genes, *Gli1* and *Ptch1*, are increased, which protects intramuscular adipogenesis of FAPs. Blocking FAP ciliation also enhances myofiber regeneration after injury.^[^ ^19^, ^20^ ^]^ However, how ciliary dynamics contribute to adipogenesis and myofiber repair following injury is not clear.

In visceral and subcutaneous adipose tissue, adipose progenitors (also called preadipocytes or adipocyte progenitors) are the source of adipocytes. Unlike multipotent FAPs, adipose progenitors only differentiate into adipocytes. Similar to FAPs, adipose progenitors dynamically form primary cilia during adipocyte differentiation, and cilia play a crucial role in adipogenesis.^[^ ^21^, ^22^ ^]^ We have recently shown that disruption of the ciliary dynamics of adipose progenitors downregulates their adipocyte differentiation in visceral and subcutaneous adipose tissues using trichoplein keratin filament‐binding (*Tchp*) knockout mice. *Tchp* encodes a centriolar protein that suppresses ciliogenesis at the step of ciliary axoneme extension, and its deficiency causes ciliary elongation in adipose progenitors. The elongated cilia impair the accumulation of caveolin 1 (CAV1)^+^ and GM3^+^ lipid rafts around the ciliary base where insulin‐like growth factor 1 receptor (IGF1R) is localized, suppressing insulin/Akt signaling, an essential signal transduction pathway for adipogenesis.^[^ ^23^, ^24^, ^25^, ^26^, ^27^, ^28^ ^]^

In this study, we investigated the role of FAP cilia in skeletal muscle regeneration using *Tchp* ^−/−^ mice. We found that *Tchp* ^−/−^ mice displayed longer cilia in FAPs and lower intramuscular adipogenesis after injury compared with wild‐type (WT) mice. The ciliary elongation significantly restricted lipid raft‐dependent Akt activation and the adipogenic differentiation of FAPs, as observed in adipose progenitors. *Tchp* ^−/−^ mice also exhibited the facilitated myofiber regeneration, which was accompanied by enhanced infiltration of anti‐inflammatory M2 macrophages, a key event of myofiber regeneration. Most importantly, skeletal muscle FAPs produced IL13, which was promoted by the cilia‐regulated IL33/ST2/c‐Jun N‐terminal kinase 1/2 (JNK1/2) axis. Injection of IL13 alone promoted myofiber regeneration following injury. We further found that IL13 directly accelerates myoblast proliferation. Thus, our data reveal that FAPs coordinate adipogenesis and myofiber regeneration following skeletal muscle injury through cilia‐mediated insulin/Akt and ST2/JNK signaling pathways, respectively.

## 2 Results

### 2.1 TCHP Deficiency Impairs Adipogenesis Upon Injury

**Figure 1:**
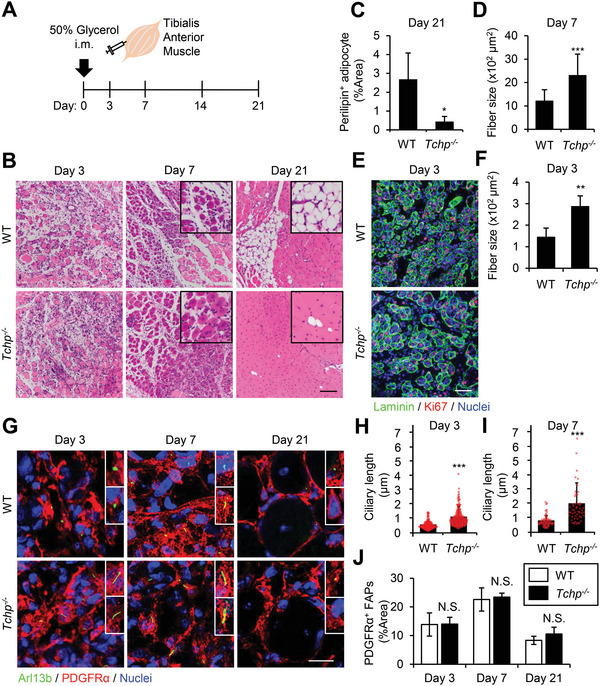
TCHP deficiency impairs adipogenesis and promotes myofiber regeneration upon skeletal muscle injury. A) Schematic of the schedule for evaluating regeneration following injury by glycerol injection into the tibialis anterior (TA) muscle of wild‐type (WT) and *Tchp^−/−^* mice. B,D) Hematoxylin and eosin staining of TA muscle cross sections at the indicated time points after injury (B) and quantification of the myofiber cross‐sectional area at 7 dpi (*n* > 100 fibers each) (D). Scale bars: 100 µm. C) Quantification of the perilipin‐stained area per 10× view at 21 dpi (*n* = three fields each). E,F) Immunofluorescence for laminin (green), Ki67 (red), and nuclei (Hoechst33342, blue) at 3 dpi (E) and quantification of myofiber cross‐sectional area (*n* = five fields each) (F). Scale bars: 50 µm. G,J) Immunofluorescence for cilia (Arl13b, green), FAPs (PDGFR*α*, red), and nuclei (Hoechst33342, blue) (G) and quantification of the PDGFR*α*‐stained area per 10× view at the indicated time points after injury (*n* = 4 to 6 fields each) (J). Scale bars: 20 µm. H,I) Scatterplot and mean bars of ciliary length at 3 dpi (*n* > 100 cells each) (H) and 7 dpi (*n* > 50 cells each) (I) evaluated from (G). All data are the mean ± S.D. from 3 to 5 mice. *p < 0.05, **p < 0.01, and ***p < 0.001, N.S., not significant; two‐tailed unpaired Student's *t*‐tests.

Given that most FAPs are dynamically ciliated during muscle regeneration,^[^ ^19^ ^]^ we examined whether TCHP deficiency influences the ciliary dynamics and adipogenic capacity of FAPs upon skeletal muscle injury. Several models of muscle injury have been described; however, we used intramuscular injection of 50% glycerol into the mouse tibialis anterior (TA) muscle because glycerol administration efficiently induces adipogenesis with transient necrosis of myofibers^[^ ^19^, ^29^ ^]^ (**Figure** **1**A). Morphological analysis by hematoxylin and eosin (H&E) staining or fat staining with perilipin demonstrated that intramuscular adipocytes started to emerge at 7 days post‐injury (dpi) in WT mice and became abundant at 14 and 21 dpi. In contrast, *Tchp^−/‐^* mice displayed a remarkable decrease in the population of adipocytes at all time points (Figure 1B,C; Figure S1A,B, Supporting Information). We also examined another muscle regeneration model, which was induced by cardiotoxin. As reported, cardiotoxin injection was less effective than glycerol injection in inducing adipogenesis^[^ ^19^, ^29^ ^]^ but far fewer adipocytes were observed in *Tchp^−/‐^* mice than in WT mice (Figure S2A,B, Supporting Information). These results indicate that *Tchp^−/−^* mice are grossly defective in adipogenesis following injury.

### 2.2 TCHP Deficiency Influences Myogenesis But Not Muscle Homeostasis

Notably, H&E‐stained tissue revealed that cross‐sectional myofiber size in *Tchp^−/‐^* mice was significantly larger than that in WT mice at 7 dpi (Figure 1B,D; Figure S1A, Supporting Information). Immunostaining of the extracellular matrix protein laminin further showed the enlargement at 3 dpi (Figure 1E,F; Figure S1C, Supporting Information). The location of myofiber nuclei gives an indication of the maturity of the fibers as the initially centrally located nuclei migrate to the periphery of regenerating fibers.^[^ ^1^ ^]^ We observed that nuclei migration also occurred sooner in *Tchp^−/‐^* mice than in WT mice (Figure S1C, Supporting Information; see 21 dpi), indicating that myofiber regeneration is accelerated in *Tchp^−/‐^* mice.

We then examined whether TCHP deficiency affects the steady state of muscle. Without injury (0 dpi), WT and *Tchp^−/‐^* mice displayed identical fiber size and peripherally located nuclei (Figure S1C,D, Supporting Information). Muscle injury and atrophy are often associated with adipose (perilipin^+^) and collagen deposition.^[^ ^30^ ^]^ Indeed, deposition of both proteins was obviously observed in glycerol‐injected WT mice at 7 dpi but rarely detected at 0 dpi in WT and *Tchp^−/‐^* mice (Figures S1A,B and S2C,D, Supporting Information). Thus, *Tchp^−/‐^* mice appear to maintain normal homeostasis of skeletal muscle in the absence of injury.

### 2.3 FAP Cilia Control Adipogenic Differentiation Via Akt Signaling

To determine whether cilia control the regenerative responses after injury and the potential underlying mechanism, we initially assessed the ciliary dynamics of FAPs in TA muscle following glycerol administration. As reported,^[^ ^19^ ^]^ WT FAPs expressing PDGFR*α* (PDGFR*α* ^+^ FAPs) transiently formed cilia, which were stained with the cilia marker, Arl13b, from 3 to 14 dpi; these cilia were then resorbed at 21 dpi (Figure 1G–I; Figure S1E, Supporting Information). In *Tchp^−/‐^* mice, PDGFR*α* ^+^ FAPs also formed cilia from 3 to 14 dpi but the cilia were longer than those in WT mice and were still present at 21 dpi. The numbers of PDGFR*α* ^+^ FAPs rapidly increased upon injury and then returned to pre‐injury level,^[^ ^8^, ^19^ ^]^ and there was no significant difference between WT and *Tchp^−/−^* mice (Figure 1J). Thus, TCHP deficiency caused FAP cilia to elongate during muscle regeneration without affecting the FAP population.

**Figure 2:**
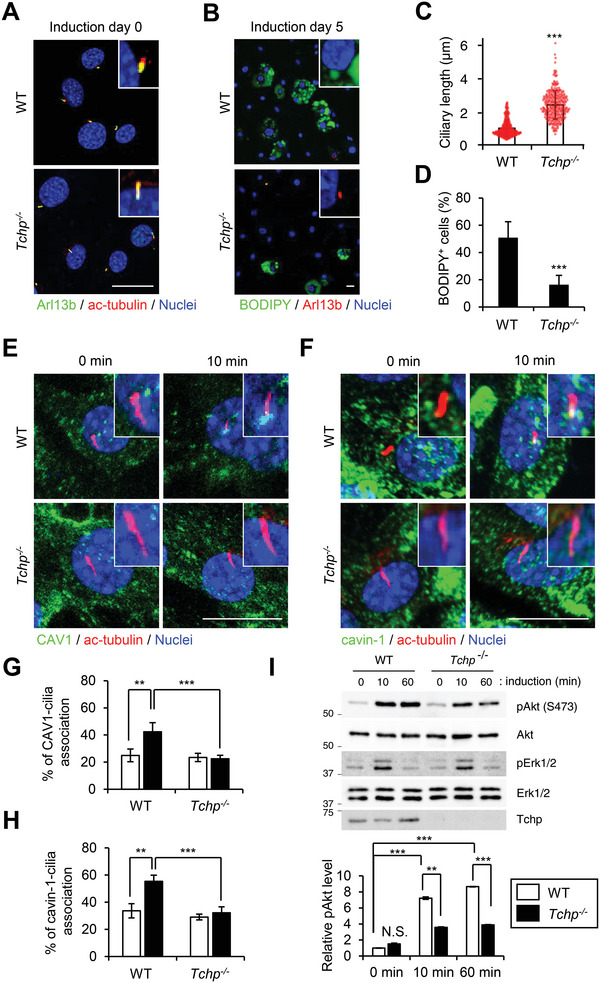
Ciliary elongation suppresses adipocyte differentiation of FAPs via Akt downregulation. PDGFR*α* ^+^ FAPs were isolated from hindlimbs of WT or *Tchp^−/−^* mice and cultured in vitro and adipogenic differentiation was induced for the indicated time periods. A,C) Immunofluorescence for cilia (Arl13b, green; acetylated‐tubulin, red) and nuclei (Hoechst33342, blue) before adipogenic induction (A) and scatterplot and mean bars of the ciliary length (*n* > 100 cells each) (C). Scale bars: 20 µm. B,D) Immunofluorescence for adipocytes (BODIPY, green), cilia (Arl13b, red), and nuclei (Hoechst33342, blue) at 5 days after adipogenic induction (B) and the percentages of BODIPY^+^ cells (*n* > 100 cells each) (D). Scale bars: 20 µm. E–H) Immunofluorescence for CAV1 (green) (E) or cavin‐1 (green) (F), cilia (acetylated‐tubulin, red) and nuclei (Hoechst33342, blue) at 0 or 10 min after adipogenic induction, and the percentage of cells that displayed an association of CAV1 (G) or cavin‐1 (H) with the ciliary base labeled with acetylated‐tubulin at 0 (white) or 10 min (black) after adipogenic induction (*n* > 50 cells each). Scale bars, 20 µm. I) Immunoblotting of whole cell lysates from samples analyzed in (E–H). Normalized mean intensities of phospho‐Akt evaluated from immunoblotting analysis are shown in a graph. All data are the mean ± S.D. from three independent replicates. **p < 0.01, ***p < 0.001, N.S., not significant; two‐tailed unpaired Student's *t*‐tests.

To examine the effect on FAP adipogenic differentiation, we isolated PDGFR*α* ^+^ FAPs from hindlimbs of WT and *Tchp^−/−^* mice and cultured them in vitro: almost all cells were labeled with FAP markers PDGFR*α* and Sca1[7, 8] (Figure S3A–C, Supporting Information). Upon plating, the WT FAPs expanded rapidly and formed cilia that stained with two distinct cilia markers, Arl13b and acetylated‐tubulin, as observed in damaged muscle; ≈50% of WT FAPs had the capacity to differentiate into BODIPY^+^ adipocytes following treatment with adipogenic stimuli, including insulin (**Figure** **2**A–D). *Tchp^−/−^* FAPs also proliferated rapidly and formed cilia but they displayed longer cilia than WT FAPs (Figure 2A,C). In addition, less than 20% of the *Tchp^−/−^* FAPs underwent adipogenic differentiation (Figure 2B,D), indicating that Tchp deficiency triggers ciliary elongation in PDGFR*α* ^+^ FAPs and suppresses their adipogenic differentiation in vitro and in vivo.

Akt plays a crucial role in the differentiation of adipose progenitors, and cilia control this signaling.^[^ ^22^ ^]^ In addition, wortmannin and Akt interfering drugs can inhibit adipogenesis of wild type and dystrophic FAPs.^[^ ^31^ ^]^ We recently showed that the insulin/Akt axis is downregulated in TCHP‐depleted adipose progenitors because the elongated cilia disrupt the accumulation of caveolin/lipid raft around the ciliary base where IGF1R is located.^[^ ^27^, ^28^ ^]^ We therefore examined the distribution of several caveolin/lipid raft markers, such as caveolin‐1 (CAV1), cavin‐1, flotillin‐2, and GM3, in PDGFR*α* ^+^ FAPs in vitro. Upon adipogenic induction, CAV1, cavin‐1, and flotillin‐2 accumulated around the ciliary base in WT FAPs but not in *Tchp^−/−^* FAPs; GM3 accumulation was not observed in FAPs from both mice (Figure 2E–H; Figure S3D–G, Supporting Information). Akt activity was also downregulated in *Tchp^−/−^* FAPs compared with WT FAPs (Figure 2I). To evaluate whether Akt activity is responsible for FAP adipogenesis, adipogenic differentiation of PDGFR*α* ^+^ FAPs was induced in the presence of the Akt inhibitors MK‐2206 and ARQ092. Both inhibitors markedly restricted FAP differentiation into BODIPY^+^ adipocytes (Figure S4, Supporting Information). These data indicate that TCHP deficiency–mediated ciliary elongation protects FAPs from adipogenic differentiation by disrupting lipid raft dynamics–dependent Akt activation, as reported in adipose progenitors.^[^ ^27^ ^]^ However, there were some differences between FAPs and adipose progenitors: flotillin‐2 accumulated at the ciliary base in FAPs but not in adipose progenitors of WT mice; conversely, GM3 accumulation was observed in adipose progenitors but not in FAPs[27] (Figure S3D–G, Supporting Information). The lipid raft dynamics around the ciliary base might be cell‐type‐ or tissue‐specific in adipogenic differentiation.

FAPs differentiate into not only adipocytes but also fibroblasts that produce collagen type 1 (Col1) and CCN family protein 2 (CCN2, also called CTGF) after injury, and transforming growth factor *β* (TGF‐*β*) plays a key role in fibrogenesis.^[^ ^32^ ^]^ To test whether TCHP deficiency influences FAP fibrogenesis, we evaluated the expression of *Col1* and *Ccn2* in PDGFR*α* ^+^ FAPs upon treatment with TGF‐*β*1 in vitro. qRT‐PCR analysis revealed that WT and *Tchp^−/−^* FAPs showed the same levels of *Col1* and *Ccn2* (Figure S5A–C, Supporting Information). Immunofluorescence analysis further showed that WT and *Tchp^−/−^* FAPs both expressed equal levels of Col1 (Figure S5D,E, Supporting Information). We then evaluated Col1 deposition in WT and *Tchp^−/−^* mice after glycerol injection. In both mice, the Col1‐stained area was the largest at 7 dpi and then decreased at 21 dpi; there were no differences between WT and *Tchp^−/−^* mice (Figure S2C,D, Supporting Information). These results indicate that TCHP deficiency restricts FAP adipogenesis but does not influence FAP fibrogenesis following injury.

### 2.4 FAPs are IL13‐Producing Cells in Skeletal Muscle Niche

We then examined the mechanism underlying the promoted muscle regeneration in *Tchp^−/−^* mice. Ectopic fat formation is generally thought to disturb muscle homeostasis and regeneration.^[^ ^32^ ^]^ Therefore, the defective adipogenesis in *Tchp^−/−^* mice might influence myofiber size following injury. However, myofiber size was already larger in *Tchp^−/‐^* mice than in WT mice at 3 dpi, before adipogenesis was detected in WT and *Tchp^−/−^* mice (Figure 1E,F; Figure S1B, Supporting Information). We therefore explored another possibility.

**Figure 3:**
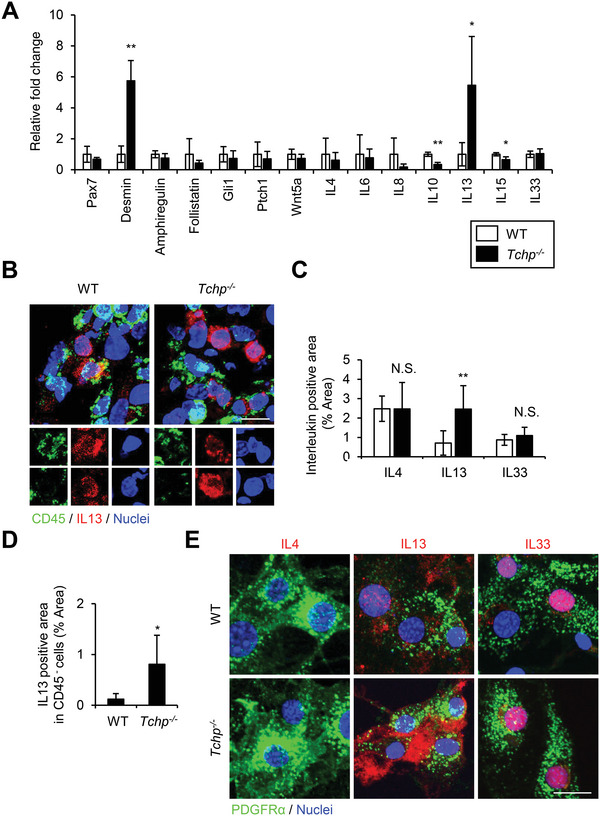
IL13 expression in FAPs is upregulated in *Tchp^−/−^* mice. A) qRT‐PCR analysis of RNA isolated from whole TA muscle of WT and *Tchp^−/−^* mice (*n* = 3 or 4) at 3 dpi. B) Immunofluorescence for IL13 (red), hematopoietic cells (CD45, green), and nuclei (Hoechst33342, blue) in TA muscle cross‐sections of WT and *Tchp^−/−^* mice at 3 dpi. Lower magnification images are shown in Figure S6A, Supporting Information. Scale bars, 10 µm. C) Quantification of the IL4‐, IL13‐, or IL33‐stained area per 40× view in (B) and Figure S6A,B (Supporting Information) (*n* = 6 or 7 fields each). D) Quantification of the IL13‐stained area per 40× view excluding the CD45‐stained area in (B) (*n* = 7 fields each). E) Immunofluorescence for IL4 (red), IL13 (red), and IL33 (red), PDGFR*α* ^+^ (green), and nuclei (Hoechst33342, blue) in PDGFR*α* ^+^ FAPs isolated from hindlimbs of WT and *Tchp* ^−/‐^ mice. Scale bars: 20 µm. All data are the mean ± S.D from 3 to 5 mice. *p < 0.05, **p < 0.01, N.S., not significant; two‐tailed unpaired Student's *t*‐tests.

We first quantified the mRNA levels of factors involved in muscle regeneration or in the function and regulation of FAPs by qRT‐PCR of whole TA muscle tissue at 3 dpi (**Figure** **3**A). The drastically elevated level of desmin, a marker for muscle differentiation in *Tchp^−/−^* mice supported the data showing accelerated regeneration of myofibers. Notably, among the cytokines that influence FAP functions (IL4, IL13, IL15, and IL33) or that are secreted from FAPs (IL6, IL10, IL33, and follistatin),^[^ ^32^, ^33^ ^]^ only IL13 mRNA level was significantly higher in *Tchp^−/−^* mice compared with WT mice.

qRT‐PCR analysis further revealed that the mRNA expression of IL15, which suppresses FAP differentiation into adipocytes following injury,^[^ ^34^ ^]^ was slightly decreased in *Tchp^−/−^* mice compared with WT mice. It is unclear why *Tchp^−/−^* muscle exhibited a decreased expression of *Il15*; the decrease seems to oppose the limited adipogenesis of *Tchp^−/−^* mice. Wnt5a, a critical non‐canonical Wnt signaling ligand, also suppresses FAP adipogenesis and participates in ciliary function^[^ ^35^ ^]^ but *Tchp^−/−^* mice demonstrated no alteration in Wnt5a mRNA levels. Mammalian Hh signal transduction depends on cilia and plays an important role in repressing injury‐induced adipogenesis;^[^ ^19^ ^]^ we evaluated the expressions of Hh target genes, *Gli1* and *Ptch1*, but found no differences between WT and *Tchp^−/−^* mice. We therefore focused on the role of IL13 in muscle regeneration.

IL13 is generally thought to be released from CD45^+^ leukocytes upon skeletal muscle injury.^[^ ^36^, ^37^, ^38^, ^39^ ^]^ However, immunofluorescence revealed that CD45^−^ non‐hematopoietic cells contained a comparable level of IL13 protein compared with CD45^+^ leukocytes in the TA muscle of WT mice at 3 dpi, and IL13 expression in *Tchp* ^−/−^ mice was higher than that in WT mice (Figure 3B–D; Figure S6A,B, Supporting Information). Moreover, IL13 was detected in PDGFR*α* ^+^ FAPs isolated from WT mice, and the protein level was greater in those derived from *Tchp* ^−/−^ mice (Figure 3E). Thus, FAPs are IL13‐expressing cells and TCHP deficiency enhances IL13 expression. As reported,^[^ ^36^, ^37^ ^]^ IL4 was only expressed in CD45^+^ leukocytes but not in PDGFR*α* ^+^ FAPs; conversely, IL33 was expressed in PDGFR*α* ^+^ FAPs but not in CD45^+^ leukocytes from skeletal muscle (Figure 3C; Figure S6A, Supporting Information).

### 2.5 Cilia Control IL13 Expression Via the ST2/JNK Axis

**Figure 4:**
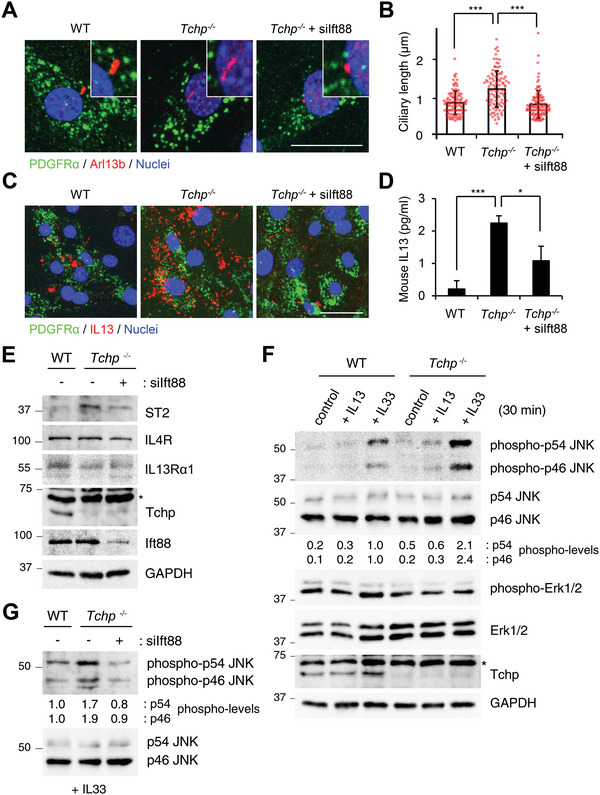
Cilia control IL13 production in FAPs in vitro. PDGFR*α* ^+^ FAPs isolated from hindlimbs of WT or *Tchp^−/−^* mice were transfected with control or *Ift88* siRNA (siift88) and cultured for 2 days. A,B) Immunofluorescence for FAPs (PDGFR*α*, green), cilia (Arl13b, red), and nuclei (Hoechst33342, blue) (A), and scatterplot and mean ± S.D. of the ciliary length (*n* > 100 cells each from three replicates) (B). Scale bar: 20 µm. C) Immunofluorescence for FAPs (PDGFR*α*, green), IL13 (red), and nuclei (Hoechst33342, blue). Scale bars: 20 µm. D) ELISA analysis of IL13 in culture media. Data are mean ± S.D. from three independent replicates. E,G) Immunoblotting of whole cell lysates from control or siift88‐transfected WT or *Tchp^−/−^* FAPs treated with (G) or without (E) IL33 (20 ng mL^−1^) for 30 min. F) Immunoblotting of whole cell lysates from WT or *Tchp^−/−^* FAPs treated with PBS (control), IL13 (20 ng mL^−1^), or IL33 (20 ng mL^−1^) for 30 min. Normalized mean intensities of phospho‐p54/p46 JNK are shown. Asterisks indicate non‐specific bands. *p < 0.05, ***p < 0.001, N.S., not significant; two‐tailed unpaired Student's *t*‐tests.

To determine whether FAP cilia regulate IL13 expression, we depleted intraflagellar transport protein 88 (IFT88), which is involved in primary cilia biogenesis and maintenance, from PDGFR*α* ^+^ FAPs isolated from *Tchp^−/−^* mice by siRNA. Two days after transfection with *Ift88* siRNA, the length of cilia was restored to the length in PDGFR*α* ^+^ FAPs isolated from WT mice (**Figure** **4**A,B). Immunofluorescence analysis revealed that the higher level of intracellular IL13 in *Tchp* ^−/−^ FAPs was decreased by IFT88 knockdown (Figure 4C). ELISA analysis of culture medium further showed that the secreted levels of IL13 correlated with the intracellular levels (Figure 4D). Thus, cilia have a crucial role in IL13 release from skeletal muscle FAPs.

How do FAP cilia regulate IL13 production? We focused on the IL33 signaling because IL33 induces IL13 expression in other cell types.^[^ ^40^, ^41^, ^42^ ^]^ We found that the expression of ST2, an IL33 receptor, was elevated by TCHP deficiency and rescued by co‐depletion of TCHP and IFT88 (Figure 4E). IL4 receptor (IL4R) and IL13 receptor *α*1 (IL13R*α*1) are also expressed in FAPs^[^ ^43^ ^]^ but their expressions were not influenced by TCHP deficiency or IFT88 depletion.

Treatment of PDGFR*α* ^+^ FAPs with IL33 activated c‐jun N‐terminal kinase 1/2 (JNK1/2) but not ERK1/2, among the downstream effectors of the IL33/ST2 axis;^[^ ^44^ ^]^ the IL33‐induced JNK1/2 activation was greater in *Tchp^−/‐^* FAPs than in WT FAPs (Figure 4F), consistent with their ST2 expression levels (Figure 4E). Furthermore, the enhanced JNK1/2 activity in *Tchp^−/−^* FAPs was reversed by IFT88 knockdown (Figure 4G), indicating that ciliary length is critical for ST2 expression and JNK1/2 activity in FAPs.

**Figure 5:**
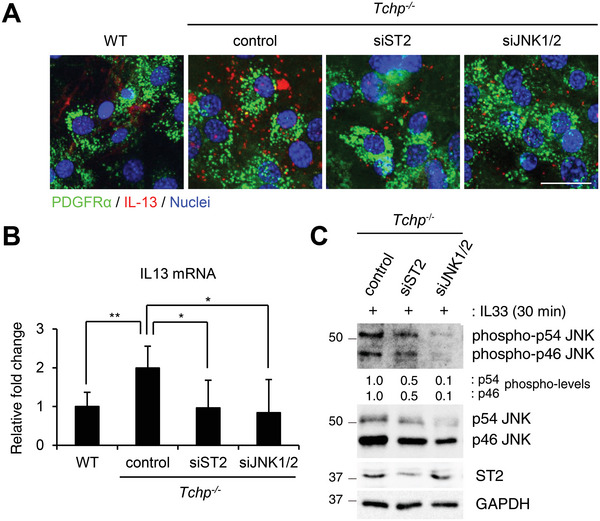
ST2/JNK signaling controls IL13 production by FAPs in vitro. PDGFR*α* ^+^ FAPs isolated from the hindlimbs of WT or *Tchp^−/−^* mice were transfected with control siRNA, siRNA targeting ST2 (siST2) or siRNA targeting JNK1 plus JNK2 (siJNK1/2) and cultured for 2 days. A) Immunofluorescence for FAPs (PDGFR*α*, green), IL13 (red), and nuclei (Hoechst33342, blue). Scale bars, 20 µm. B) qRT‐PCR for *Il13* from RNA isolated from FAPs (*n* = 3). Data are the mean ± S.D. ***p < 0.001; two‐tailed unpaired Student's *t*‐tests. C) Immunoblotting of whole cell lysates from siRNA‐transfected *Tchp^−/−^* FAPs treated with IL33 (20 ng mL^−1^) for 30 min. Normalized mean intensities of phospho‐p54/p46 JNK are shown.

We then tested whether the elevated ST2/JNK1/2 pathway is responsible for the increased IL13 production in *Tchp^−/−^* FAPs. Both immunofluorescence and qRT‐PCR analyses revealed that either knockdown of ST2 or JNK1/2 decreased IL13 production (**Figure** **5**A,B). ST2 knockdown also downregulated JNK1/2 activity (Figure 5C). FAP cilia also participated in Akt or Hh signaling; and thus, we examined their involvement in IL13 production using chemical inhibitors. Neither MK‐2206 (an Akt inhibitor) nor GANT61 (a GLI inhibitor) influenced the intracellular level of IL13 in WT and *Tchp^−/−^* FAPs (Figure S6C,D, Supporting Information), indicating that FAP cilia control IL13 production through the IL33/ST2/JNK axis.

IL33 released from FAPs also recruits FOXP3^+^ Tregs to damaged muscle tissue and then Tregs secrete amphiregulin to promote muscle regeneration.^[^ ^11^, ^13^ ^]^ We therefore examined whether TCHP deficiency influences Treg accumulation. Injection of glycerol and cardiotoxin have distinct impacts on Treg accumulation; intramuscular injection of cardiotoxin triggers effective Treg accumulation, while glycerol injection has little effect.^[^ ^13^ ^]^ However, for both treatments, there was no significant difference in Treg accumulation between WT and *Tchp^−/−^* mice (Figure S7, Supporting Information). These results are consistent with the comparable levels of amphiregulin produced in glycerol‐injected WT and *Tchp^−/−^* mice at 3 dpi (Figure 3A). Therefore, FAP cilia are not related to Treg function in muscle regeneration.

### 2.6 IL13 Promotes M2 Polarization

Muscle regeneration relies upon the coordinated actions of macrophages. Following injury, resting, M0‐type, macrophages are polarized into different phenotypes: the pro‐inflammatory M1‐type (M1 polarization) or anti‐inflammatory M2‐type (M2 polarization). These macrophages perform different roles. M1 macrophages are the first to appear at damaged muscle and clear apoptotic or necrotic cells and tissue debris through efferocytosis. M2 macrophages subsequently become dominant and orchestrate muscle regeneration.^[^ ^4^, ^5^ ^]^

**Figure 6:**
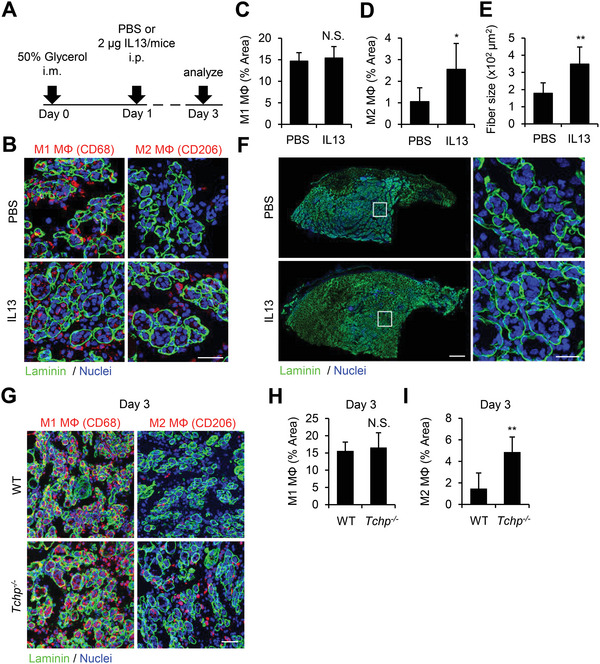
IL13 promotes muscle regeneration and M2 polarization. IL13 or PBS was intraperitoneally injected into glycerol‐injected WT mice at 1 dpi. A) Schematic of the experimental schedule. B) Immunofluorescence analysis of CD68^+^ M1 (M1 MΦ, red) or CD206^+^ M2 (M2 MΦ, red), laminin (green), and nuclei (Hoechst33342, blue) in TA muscle cross sections at 3 dpi. C,D) Quantification of the CD68‐stained area (*n* = 5 or 6) (C) or the CD206‐stained area (*n* = 8) (D) per 40× view in (B). TA muscle cross‐sections of PBS‐ or IL13‐injected mice at 3 dpi. Scale bars, 40 µm. E,F) Quantification of average cross‐section area of myofiber in TA muscle of PBS‐ (*n* = 5) or IL13‐injected mice (*n* = 8) at 3 dpi (E) and representative images of immunofluorescence for laminin (green) and nuclei (Hoechst33342, blue) (F). Scale bars, 400 µm (left) and 20 µm (right). G–I) Immunofluorescence analysis of CD68^+^ M1 (M1 MΦ, red) or CD206^+^ M2 (M2 MΦ, red), laminin (green), and nuclei (Hoechst33342, blue) in TA muscle cross sections of glycerol‐injected WT and *Tchp^−/−^* mice at 3 dpi (G) and quantification of CD68‐stained area (*n* = 5 or 6) (H) or CD206‐stained area (*n* = 8) (I) per 40× view. Scale bar: 50 µm. All data are the mean ± S.D. *p < 0.05, **p < 0.01, N.S., not significant; two‐tailed unpaired Student's *t*‐tests.

IL13 has an important role in M2 polarization. Therefore, we examined the effect of IL13 during muscle regeneration in vivo. We intraperitoneally injected recombinant mouse IL13 or PBS into glycerol‐injected WT mice at 1 dpi (**Figure** **6**A). As expected, IL13 injection facilitated the infiltration of CD206^+^ M2 macrophages but not CD68^+^ M1 macrophages, at 3 dpi compared with results in PBS‐injected mice (Figure 6B–D). Intriguingly, myofiber size was significantly enlarged in IL13‐injected WT mice compared with PBS‐injected WT mice (Figure 6E,F), as observed in *Tchp^−/−^* mice (Figure 1E,F). Thus, we examined the effect of TCHP depletion on macrophage polarization after injury. We found that *Tchp^−/−^* mice exhibited the increased infiltration of CD206^+^ M2 macrophages but not CD68^+^ M1 macrophages, at 3 dpi compared with WT mice without IL13 injection (Figure 6G–I). Given the higher IL13 level of *Tchp^−/−^* mice at 3 dpi (Figure 3A), the promoted M2 polarization is likely to be caused by the increased IL13 in *Tchp^−/−^* mice.

*Tchp* expression was detected in macrophages of WT but not *Tchp^−/−^* mice (Figure S8A, Supporting Information), raising the possibility that macrophage‐intrinsic trichoplein influences M2 polarization. To exclude this possibility, we tested whether TCHP deficiency influences macrophage polarization in vitro. We purified mouse monocytes using magnetic beads, differentiated them into M0 macrophages by cultivation with macrophage colony stimulating factor (M‐CSF), and polarized them into M1 (TNF*α* ^+^, IL6^+^) or M2 (Mrc1^+^, Arg1^+^) macrophages by cultivation with interferon *γ* (IFN*γ*) and lipopolysaccharide (LPS) or with IL4 and IL13, respectively.^[^ ^45^, ^46^, ^47^ ^]^ Assessment of M1 and M2 macrophage markers by qRT‐PCR revealed that WT and *Tchp^−/−^* mouse‐derived M0 macrophages were identically polarized into M1 or M2 macrophages by each treatment (Figure S8B–E, Supporting Information), indicating that macrophage‐intrinsic trichoplein seems to not participate in macrophage polarization.

### 2.7 IL13 Promotes Myofiber Regeneration

**Figure 7:**
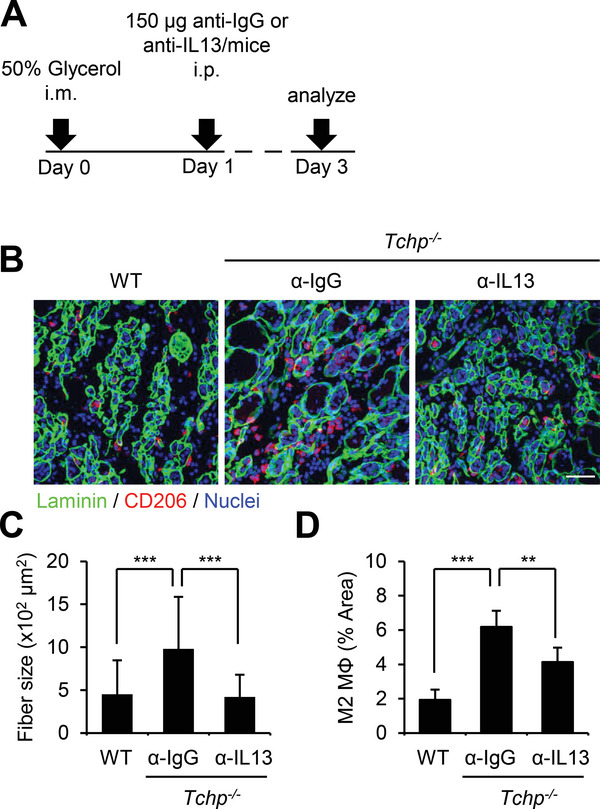
Anti‐IL13 neutralizing antibody ameliorates the phenotypes of glycerol‐injected *Tchp* ^−/−^ mice. Anti‐IL13 neutralizing or control anti‐IgG antibody was injected into glycerol‐injected WT or *Tchp* ^−/−^ mice at 1 dpi. A) Schematic of the experimental schedule. B) Representative immunofluorescence images of laminin (green), CD206 (M2 MΦ, red), and nuclei (Hoechst33342, blue) in TA muscle cross sections at 3 dpi. Scale bars, 20 µm. C,D) Quantification of average cross‐section area of myofiber size (C) and CD206‐stained area per 40× view (D) in TA muscle cross sections of anti‐IgG‐injected WT (*n* = 6) and *Tchp* ^−/−^ (*n* = 6) mice and anti‐IL13‐injected *Tchp* ^−/−^ mice (*n* = 6) at 3 dpi. All data are the mean ± S.D. **p < 0.01, ***p < 0.001; two‐tailed unpaired Student's *t*‐tests.

The close resemblance in phenotypes of IL13‐injected WT and *Tchp^−/−^* mice indicates that excess IL13 might be a major cause of the promoted muscle regeneration. To verify this hypothesis, we tested whether a neutralizing antibody against IL13 would ameliorate the phenotypes of *Tchp^−/−^* mice. We intraperitoneally injected anti‐IL13 or control anti‐IgG antibody into glycerol‐injected *Tchp^−/−^* mice at 1 dpi (**Figure** **7**A). As shown above, control *Tchp^−/−^* mice displayed enlarged myofiber size and promoted M2 polarization at 3 dpi compared with control WT mice; however, both phenotypes were significantly reverted in *Tchp^−/−^* mice injected with anti‐IL13 antibody (Figure 7B–D). Thus, higher IL13 level is a bona fide cause of accelerated myofiber regeneration and increased M2 polarization of *Tchp^−/−^* mice after injury.

### 2.8 IL13 Promotes Myoblast Proliferation

While IL13‐mediated M2 polarization has been established,^[^ ^48^ ^]^ how IL13 increases myofiber size upon injury is unknown. IL13 shares many biological properties with IL4, stemming from the fact that they share a common receptor subunit, IL4R. Therefore, IL13, like IL4, is thought to facilitate muscle repair by enhancing FAP proliferation through stimulation of signal transducer and activator of transcription 6 (STAT6).^[^ ^43^ ^]^ However, neither TCHP deficiency (Figure 1J) nor IL13 injection (Figure S9A–C, Supporting Information) influenced the FAP population following injury. We further confirmed that in vitro treatment of PDGFR*α* ^+^ FAPs with IL13 had no effect on their number (Figure S9D, Supporting Information). Thus, unlike IL4, IL13 is not involved in proliferation of FAPs.

**Figure 8:**
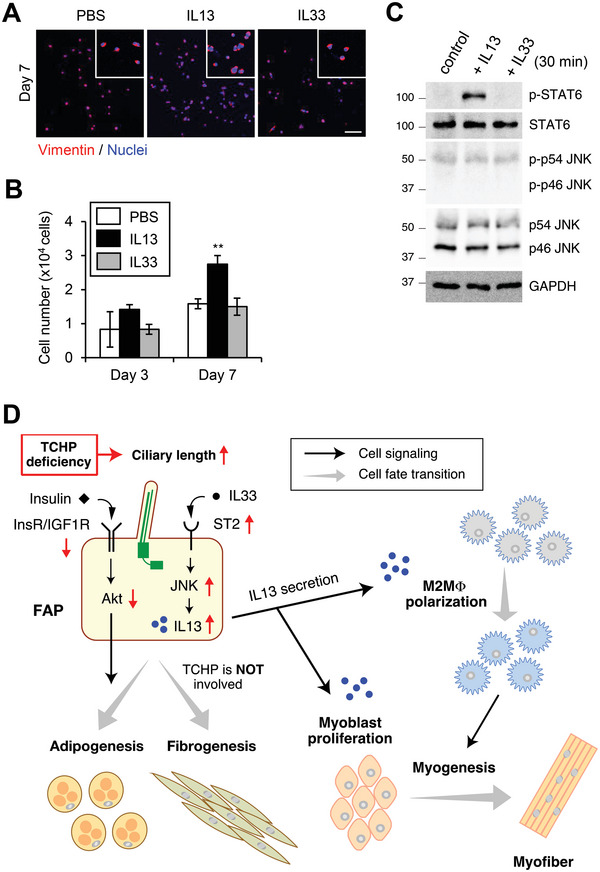
IL13 directly promotes myoblast proliferation. Primary myoblasts isolated from hindlimbs of WT mice were cultured in growth medium containing PBS (control), IL13 (20 ng mL^−1^) or IL33 (20 ng mL^−1^). A) Representative images of immunofluorescence for vimentin (myoblast marker, red) and nuclei (Hoechst33342, blue) at 7 days. Scale bars, 100 µm. B) Quantification of the number of primary myoblasts at 3 or 7 days. Data are the mean ± S.D. from three mice. **p < 0.01, two‐tailed unpaired Student's *t*‐tests. C) Immunoblotting of whole cell lysates from primary myoblasts treated with PBS, IL13, or IL33 for 30 min. D) Proposed model. FAP cilia control two distinct signaling pathways involved in muscle recovery, insulin/Akt, and IL33/ST2/JNK signaling, which are down‐regulated or up‐regulated by TCHP deficiency–induced ciliary elongation, respectively. Downregulation of insulin/Akt signaling restricts FAP adipogenesis. Upregulation of IL33/ST2/JNK signaling increases IL13 secretion from FAPs, which promotes myoblast proliferation and M2 polarization, enhancing myofiber regeneration. Signaling up‐ or down‐regulation induced by TCHP depletion is shown by red arrows. InsR, insulin receptor; M2MΦ, M2 macrophage.

To explore a novel target of IL13, we verified the effect of IL13 in vitro because the regenerative processes are complexly intertwined with related factors in vivo. IL13 directly enhanced the proliferation of primary myoblasts isolated from the hindlimbs of WT mice and stimulated STAT6 phosphorylation (**Figure** **8**A–C). Myoblasts did not respond to IL33 stimulation.

We next checked whether muscle intrinsic trichoplein is involved in the proliferation and differentiation of satellite cells or myoblasts using ex vivo and in vitro culture systems. We first isolated single muscle fibers, which contained PAX7^+^ satellite cells, from the extensor digitorum longus muscle of WT and *Tchp^−/−^* mice and cultured them in growth medium.^[^ ^49^ ^]^ After culture for 3 days, both WT and *Tchp^−/−^* satellite cells were labeled with Ki67, a proliferation marker, and underwent clonal expansion ex vivo (Figure S10A, Supporting Information). We next isolated PAX7^+^ primary myoblasts from hindlimbs of WT and *Tchp^−/‐^* mice; these cells contained almost identical populations of Ki67^+^ cells and mitotic cells (judged by histone H3 Ser10 phosphorylation) that exhibited similar growth in vitro (Figure S10B–E, Supporting Information). Upon myogenic induction, these myoblasts transited from the proliferative state (vimentin^+^) to the differentiated state (desmin^+^).^[^ ^50^, ^51^ ^]^ Morphological examination confirmed this transition. There was no difference between the WT and *Tchp^−/−^* myoblasts (Figure S10F–I, Supporting Information). Taken together, these results indicate that TCHP depletion from satellite cells or myoblasts has no effect on their proliferation or differentiation.

These results collectively indicate that FAP cilia control IL13 production through the IL33/ST2/JNK axis and that IL13 has key roles in muscle regeneration not only by inducing M2 polarization but also by promoting myoblast proliferation (Figure 8D).

### 2.9 IL13 Has No Significant Effect on Adipogenesis and Fibrogenesis

To assess the role of IL13 in FAP differentiation into adipocytes and fibroblasts, we examined the deposition of adipose (perilipin^+^) and collagen (Col1^+^) in PBS‐ and IL13‐injected mice at 3 and 7 dpi following glycerol injection (Figure S11A, Supporting Information). In contrast to *Tchp^−/−^* mice, IL13‐injected mice displayed no change in adipose deposition (Figure S11B, C, Supporting Information), indicating that FAP cilia control FAP adipogenesis through insulin/Akt signaling but not ST2/JNK signaling. There was no difference in collagen deposition between PBS‐ and IL13‐injected mice or between WT and *Tchp^−/−^* mice (Figure S11D, E, Supporting Information), indicating no involvement of FAP cilia in fibrogenesis. We further examined the effect of IL13 using PDGFR*α* ^+^ FAPs in vitro. As expected, IL13 showed no impact on the TGF‐*β*1‐induced fibrogenic differentiation (Figure S12A–C, Supporting Information). However, treatment of PDGFR*α* ^+^ FAPs with IL13 somewhat suppressed their adipocyte differentiation in vitro (Figure S12D,E, Supporting Information), indicating we cannot completely rule out an effect of IL13 on adipogenesis. However, our data at least indicate that IL13 directly promotes muscle regeneration without being associated with the impaired adipogenesis of FAPs following injury.

## 3 Discussion

In this study, we found that TCHP depletion induced the ciliary elongation of intramuscular FAPs following injury (Figure 1G,H). The elongated phenotype was often caused by dysregulation of ciliary proteins and caused cilia‐related disorders, such as Meckel–Gruber syndrome, through the disruption of proper ciliary dynamics and function.^[^ ^52^ ^]^ We used *Tchp^−/−^* mice to investigate the functions of FAP cilia during muscle regeneration and demonstrated that *Tchp^−/−^* mice were protected from adipogenesis following injury (Figure 1B,C). A similar phenotype was observed in FAP^no cilia^ mice, in which injury‐induced ciliogenesis was blocked in FAPs,^[^ ^19^ ^]^ indicating that properly controlled ciliary dynamics is required for FAPs to differentiate into adipocytes. Hh signaling depends on cilia; therefore, FAP^no cilia^ mice exhibit dysregulation of the Hh signaling pathway, which restricts adipogenic differentiation.^[^ ^19^ ^]^ In contrast, TCHP depletion–induced ciliary elongation disturbed the accumulation of caveolin/lipid rafts around the ciliary base, which suppressed insulin/Akt signaling, a critical signaling for adipogenesis (Figure 2E–I), without affecting Hh signal transduction (Figure 3A). IGF‐1 secreted by various muscle resident cells functions as an important myokine/cytokine in skeletal muscle maintenance and regeneration.^[^ ^53^ ^]^ TCHP deficiency had no impact on collagen deposition following injury and FAP differentiation into fibroblasts (Figures S2C and S5, Supporting Information). These data indicate that the ciliary phenotype induced by TCHP depletion restricts FAP adipogenesis but does not influence FAP fibrogenesis during muscle regeneration through cilia‐dependent signaling pathways, including the insulin/Akt pathway (Figure 8D).

FAPs also participate in myofiber repair by promoting satellite cell proliferation and M2 polarization through the secretion of several cytokines.^[^ ^8^, ^11^, ^30^, ^32^, ^33^, ^54^ ^]^ Here, we report that both myofiber regeneration and M2 polarization were facilitated in *Tchp^−/−^* mice following injury (Figures 1E,F and 6G). However, none of the known cytokines released from FAPs showed changes in mRNA levels (Figure 3A). Importantly, we identified FAPs as IL13‐producing cells and found that IL13 expression was enhanced by TCHP depletion in a ciliary length–dependent manner (Figures 3B–E and 4A–D). Injection of IL13 alone facilitated both expansion of myofiber size and M2 polarization in glycerol‐injected WT mice (Figure 6), as observed in glycerol‐injected *Tchp^−/−^* mice. Conversely, injection of anti‐IL13 neutralizing antibody restricted these phenomena. Thus, we conclude that an excess of IL13, which is induced by TCHP depletion–mediated ciliary elongation, promotes myofiber regeneration and M2 polarization (Figure 8D).

M2 polarization induced by IL13 stimulation supports myogenesis by the initial secretion of high levels of IGF‐1, which promote satellite cell proliferation, and then low levels of TNF*α* and TGF*β*, which facilitate myogenic differentiation.^[^ ^30^ ^]^ In contrast, we found that IL13 directly stimulated STAT6 phosphorylation of myoblasts and promoted their proliferation (Figure 8A–C). Given their similarities, IL13, like IL4, is thought to facilitate proliferation of FAPs^[^ ^43^ ^]^ but our results showed that IL13 did not enhance FAP proliferation both in vitro and in vivo (Figure S9, Supporting Information). Thus, IL13 participates in muscle regeneration through at least two mechanisms, by enhancing M2 polarization and by accelerating myoblast proliferation (Figure 8D).

Our conclusions have been drawn mainly using pan *Tchp* knockout mice, raising the possibility that other mechanisms may be involved in the effects of TCHP deficiency on myofiber regeneration. Our in vitro analysis revealed that TCHP depletion from macrophages had no impact on their polarization (Figure S8, Supporting Information). In addition, WT and *Tchp* ^−/−^ muscle cells, including satellite cells and myoblasts, showed no differences in proliferation and differentiation (Figure S10, Supporting Information). Although we cannot exclude the commitment of other muscle resident cells, such as endothelial cells or pericytes, injection of anti‐IL13 neutralizing antibody significantly ameliorated the promoted myofiber regeneration of glycerol‐injected *Tchp^−/−^* mice, indicating that IL13 plays a crucial role in the process of myofiber regeneration. A conditional depletion of *Tchp* only in FAPs will resolve this issue.

How do cilia regulate IL13 production? We showed that skeletal muscle FAPs express ST2 and that this expression was enhanced in *Tchp^−/−^* mice in a cilia‐dependent fashion (Figure 4E). IL33 treatment of FAPs directly activated JNK1/2, a downstream effector of the IL33/ST2 axis,^[^ ^44^ ^]^ and its activation was potentiated by TCHP deficiency (Figure 4F,G). Moreover, either knockdown of ST2 or knockdown of JNK1/2 downregulated IL13 in *Tchp^−/−^* FAPs (Figure 5), indicating that the IL33/ST2/JNK axis upregulates IL13 expression to facilitate myofiber regeneration. Hh signaling relates IL33‐induced proliferation in other cell types,^[^ ^41^, ^55^ ^]^ THCP deficiency showed no influence on the expression of Hh signaling target genes *Gli* and *Ptch1* (Figure 3A). Further experiments will be required to unveil the mechanism by which cilia regulate ST2 expression.

In summary, this work reveals that primary cilia on FAPs have crucial roles in the differentiation of FAPs into adipocytes by regulating the insulin/Akt axis and in muscle regeneration by regulating IL13 secretion via the ST2/JNK axis. These findings expand our understanding of the molecular mechanisms by which cilia orchestrate muscle regeneration and of the pathophysiological mechanisms underlying muscle diseases and other clinical phenotypes caused by ciliary deficits.

## 4 Experimental Section

### Animals

All animal experiments in this study were performed in accordance with the guidelines of Mie University Committee for Animal and Recombinant Experiments. C57BL/6J mice were purchased from Japan CLEA. *Tchp^−/‐^* mice were generated as described in a previous report.^[^ ^27^ ^]^ The investigator performed the mice experiments approved by the Institutional Animal Care and Use Committee of Mie University (approval no. 28‐18).

For muscle injury, 50 µL of 50% glycerol or 10 µm cardiotoxin was administrated to the left TA muscle, and saline was administered to the contralateral TA muscle as a control. For IL13 administration, 2 µg of recombinant mouse IL13 or control saline was injected intraperitoneally at 1 or 4 dpi. Following injury, the TA muscle was collected at the indicated time points (0, 3, 7, 14, or 21 dpi). For neutralization of IL13, 150 µg of mouse monoclonal antibody (eBio1316H, eBioscience) or control IgG*κ* (eBRG, eBioscience) was injected intraperitoneally at 1 dpi after glycerol injection.

### Primary FAP and Myoblast Isolation

Primary FAPs and myoblasts were isolated as described previously.^[^ ^49^, ^50^, ^51^ ^]^ Briefly, hindlimb muscle was dissected from 4‐ or 5‐week‐old mice and minced finely by scissors. The tissues were treated with Dispase II for 5 min and then shaken at 250 rpm for 15 min. Samples were treated with collagenase type II for 3 min and then shaken at 250 rpm for 10 min. Enzyme treatments were performed at 37 °C. The enzyme‐treated tissues were filtered through a 70‐µm pore‐size filter, washed with RBC lysis buffer, and filtered through a 35‐µm pore‐size filter. The filtrates were pre‐cultured in a polystyrene 10‐cm culture dish with DMEM high glucose medium supplemented with 10% FBS and penicillin/streptomycin (P/S) for 90 min. For isolation of FAPs, the culture medium was replaced with new medium containing 10 ng mL^−1^ human bFGF. For the isolation of myoblasts, the supernatant following pre‐culture was centrifuged at 1300 rpm for 3 min and the cell pellet was cultured on 0.67% gelatin–coated 6‐well‐plates with F‐10 medium supplemented with 20% horse serum, 5 ng mL^−1^ human bFGF, and P/S. The culture medium was changed every other day.

### Isolation of Single Fibers From EDL Muscle

EDL muscles were isolated from 6 to 8‐week‐old mice, as described previously.^[^ ^49^ ^]^ Briefly, two EDL muscles were collected from one mouse and the muscles were treated with 0.2% collagenase type I in DMEM high glucose, sodium pyruvate medium at 37 °C for 1–2 h. The samples were then suspended by pipetting up and down with a 200–1000 µL sterile tip and then, single fibers were transferred to pre‐warmed DMEM high glucose, sodium pyruvate medium. To remove debris, the procedure was repeated at least three times. After the incubation at 37 °C for 1 h, the fibers were sampled immediately (as the day 0 samples of Figure S10A, Supporting Information) or were cultured with DMEM high glucose, sodium pyruvate medium supplemented with 20% FBS, 1% chicken embryo extract, and P/S for 3 days (as the day 3 samples of Figure S10A, Supporting Information).

### Cell and Muscle Fiber Culture

Primary FAPs were cultured in DMEM high glucose medium supplemented with 10% FBS, human basic FGF and P/S and used for experiments within two passages. For adipogenic differentiation, FAPs were initially incubated with induction medium (DMEM high glucose supplemented with 200 µm indomethacin, 0.5 mm 3‐isobutyl‐1‐methylxanthine (IBMX), 1 µm dexamethasone, and 10 µg mL^−1^ human insulin) for 3 days and then with differentiation medium (DMEM high glucose supplemented with 10 µg mL^−1^ human insulin) for 2 days. In experiments with Akt inhibition, Akt kinase inhibitors, MK‐2206 (Selleck) or ARQ 092 (Selleck), were added into culture medium 30 min before adipogenic stimulation and were left in medium during adipogenic differentiation. For fibrogenic differentiation, FAPs were cultured with growth medium supplemented with 5 ng mL^−1^ mouse TGF‐*β*1 for 3 days. For stimulation or inhibition of Hh signal transduction, FAPs were treated with 500 nm smoothened agonist SAG (ab142160, Abcam) or 20 µm Gli1 inhibitor GANT1 (Selleck), respectively, for 12 h.

Primary myoblasts were cultured in F‐10 medium supplemented with 20% horse serum, 5 ng mL^−1^ human basic FGF and P/S and used for experiments within five passages. For differentiation of myoblasts, the cells were incubated with F‐10 medium supplemented with 2% horse serum and P/S. For growth assay of myoblasts, cells were seeded in 6‐well‐plates (1 × 10^4^ cells per well) and the cell number was counted with a hemocytometer at 3 or 7 days after seeding.

SiRNA transfection was performed with Lipofectamine RNAiMAX (Invitrogen). The sequences of siRNA are as follows: control siRNA (AATTCTCCGAACGTGTCACGT), mouse ift88 siRNA #1 (AAGGCATTAGATACTTATAAA), mouse Il1rl1 siRNA #1 (AACGTGACTCATGATGATGAA), mouse Mapk8 siRNA #1 (CAGGCCTAAATACGCTGGATA), and mouse Mapk9 siRNA #1 (CAGATCCTGATCTGTAAATTA) (QIAGEN, Hilden, Germany).

### Macrophage Polarization

Bone marrow cells were collected from mouse femurs. After RBC lysis, monocytes labeled with APC anti‐mouse F4/80 (BM8, Biolegend) were isolated using anti‐APC MicroBeads (Miltenyi Biotec) and an LS Column (Miltenyi Biotec). F4/80^+^ monocytes (1 × 10^6^) were cultured in DMEM supplemented with 10% FBS and 20 ng mL^−1^ recombinant mouse M‐CSF (Biolegend) for 3 days and then the medium was changed with the fresh medium for culture for a further 2 days. The medium was changed to 10% FBS‐containing DMEM supplemented with 100 ng mL^−1^ recombinant mouse IFN‐*γ* (Biolegend) and 100 ng mL^−1^ lipopolysaccharide (LPS) from *E. coli* O111 (Fujifilm WAKO) for M1 polarization; 20 ng mL^−1^ recombinant mouse IL4 (Biolegend) and 20 ng mL^−1^ recombinant mouse IL13 (Biolegend) for M2 polarization; or PBS for M0 macrophage preparation. Two days after treatment, macrophages were collected and analyzed.^[^ ^45^, ^46^, ^47^ ^]^

### Immunohistochemistry and Immunocytochemistry

Mouse TA muscles were prepared from 8 to 12‐weeks‐old WT or *Tchp^−/‐^* mice and incubated in 4% paraformaldehyde/PBS overnight. For hematoxylin and eosin staining, the fixed tissues were embedded in paraffin wax. For immunocytochemistry, the fixed tissues were treated with PBS, 15% and 30% sucrose/PBS, embedded with OCT compound and stored at 80 °C. Sections (10 µm thick) were sliced by a cryostat (Leica, CM1860). Staining was performed using the following antibodies: acetylated tubulin (6‐11B‐1, Sigma–Aldrich; 1:200), Arl13b (17711‐1‐AP, Proteintech; 1:200), PTRF (18892‐1‐AP, Proteintech; 1:100), Caveolin‐1 (#3238, Cell Signaling Technology; 1:300), Collagen I (ab21286, Abcam; 1:100), Perilipin (D1D8, Cell Signaling Technology; 1:200), CD45 and CD45‐biotin (30‐F11, Biolegend; 1:100), CD68 (FA‐11, Bio‐Rad; 1:100), CD206 (MR5D3, Bio‐Rad; 1:100), Desmin (D93F5, Cell Signaling Technology; 1:200), Flotillin‐2 (B‐6, Santa Cruz Biotechnology; 1:20), FOXP3 (FJK‐16s, Invitrogen; 1:100), GM3 (GMR6, Tokyo Chemical Industry; 1:500), IL4 (BVD4‐1D11, Novus Biologicals; 1:100), IL13 (AF‐413, R&D Systems; 1:100), IL33 (AF3626, R&D Systems; 1:100), PAX7 (MAB1675, R&D Systems; 1:100), PDGFR*α* (AF1062, R&D Systems; 1:200; APA5, Biolegend; 1:100), p‐Histone H3 (Ser 10) (sc‐8656‐R, Santa Cruz Biotechnology; 1:100), Sca1 (E13‐161.7, Biolegend, 1:100), Vimentin (#280618, R&D Systems; 1:200), Ki67 (SolA15, Invitrogen; 1:200), and Laminin (L9393, Sigma Aldrich; 1:200). Secondary antibodies were Alexa Fluor 488, 555, 647, or Cy3‐conjugated IgG (1:200) (Jackson Laboratories). For IL13, IL33, or PDGFR*α* antibodies (goat IgG), blocking solution supplemented with normal donkey serum and donkey IgG secondary antibodies was used. For other antibodies, blocking solution containing normal goat serum and secondary antibodies was goat IgG. Secondary antibodies were used at 1:200 dilution. Nuclei were stained by Hoechst33342 (1:1000) (DOJINDO). For PDGFR*α* staining, antigen‐retrieval treatment was performed using pepsin at 37 °C at 30 min before blocking. In double staining using rat‐derived IL4 and CD45‐biotin antibodies, samples were incubated first with IL4 antibody and anti‐rat IgG‐conjugated Alexa Fluor 488 as a secondary antibody. For flotillin‐2 staining, cells were fixed/permeabilized by the methanol/acetone method. All fluorescence images were collected as Z‐stack images using confocal microscopy FV‐1000 (Olympus) using UPlanSApo10×/0.40, UPlanSApo20×/0.75, UPlanSApo40×/0.95 or UPlanSApo60×/1.35oil.

For measurement of ciliary length, confocal Z‐stack images of sections stained with acetylated tubulin or Arl13b were analyzed by Image J software (Fiji, 1.53f51); measurements were taken from the base to the tip of the axoneme. The measured values do not necessarily demonstrate the exact values of individual ciliary length because cilia grow not only toward the *xy* plane but also toward random direction, especially in TA muscle cross‐sections. Thus, the data are shown not only as the mean ± S.D. but also as the scatter plots, which represent the frequency of ciliary elongation. For calculation of percentage area, Z‐stack images of sections stained with the indicated antibodies were analyzed with Image J using the “Threshold” function.

### SDS‐PAGE and Western Blotting

Cells were lysed with RIPA lysis buffer and incubated on ice for 30 min. After centrifugation at 15 000 rpm for 5 min, supernatants were collected and mixed with 6× SDS sample buffer. The samples were boiled at 95 °C for 5 min and subjected to immunoblotting following standard procedures. The following primary antibodies were used for immunoblotting: mouse monoclonal Akt (40D4, Cell Signaling Technology; 1:1000), ERK1/2 (E10, Cell Signaling Technology; 1:1000), IL13Ralpha1 (D‐2, Santa Cruz Biotechnology; 1:1000), JNK (clone 37, BD Transduction Laboratories; 1:1000); rabbit monoclonal phospho‐Akt (Thr308) (C31E5E, Cell Signaling Technology; 1:1000), phospho‐Akt (D9E, Cell Signaling Technology; 1:1000), phospho‐ERK1/2 (D13.14.4E, Cell Signaling Technology; 1:1000), GAPDH‐HRP (14C10, Cell Signaling Technology; 1:5000), Gli1 (NB600‐600, Novus Biologicals; 1:1000), phospho‐JNK (81E11, Cell Signaling Technology; 1:1000), STAT6 (D3H4, Cell Signaling Technology; 1:1000), phospho‐STAT6 (D8S9Y, Cell Signaling Technology; 1:1000); rabbit polyclonal IFT88 (13967‐1‐AP, Proteintech; 1:1000), IL4R (bs‐2458R, Bioss; 1:1000), ST2 (ab25877, Abcam; 1:1000), and Trichoplein (home‐made; 1:1000). After staining with HRP‐conjugated secondary antibody (Molecular Probes), bands were detected using ECL chemiluminescence.

### RNA Isolation, Reverse Transcription, and Real–Time PCR

RNA was isolated from cells using the RNeasy Plus Mini Kit (Qiagen) or Monarch Total RNA Miniprep Kit (New England BioLabs). For RNA isolation from muscle tissues, 50 mg of TA muscle was used from glycerol‐injected WT and *Tchp^−/‐^* mice at 3 dpi. Muscles were homogenized using a pestle and mortar and dissolved in TRIzol Reagent (Ambion). Reverse transcription from RNA to cDNA was performed with a PrimeScript RT reagent Kit (TaKaRa). qPCR was performed with TB Green Premix Ex Taq II (Tli RNaseH Plus) (TaKaRa). Quantitative real‐time PCR was performed with QuantStudio 3 (Applied Biosystems, Waltham, MA, USA) using specific primers (Table S1, Supporting Information).

### ELISA Analysis

For quantification of mouse IL13 in culture supernatants of FAPs, the Quantikine ELISA system (R&D systems) was used. FAPs isolated from WT and *Tchp* ^−/−^ mice were cultured for 1 week and 1 × 10^5^ FAPs were re‐seeded in 12‐well‐plates with siRNA. Six hours after plating, the medium was changed with the fresh medium and incubated for 2 days. The supernatants were stored at −80 °C until ELISA analysis. The optical density of 96‐well culture plates was measured with a microplate reader Infinite F50 (TECAN).

### Statistical Analysis

Statistical analysis was performed on normally distributed data sets. In vitro and in vivo data were evaluated from at least three technical and biological replicates, respectively. Sample size is shown in the Figure legends. All data were analyzed using Microsoft Excel (Microsoft). R software was used to draw scatterplots. Data are shown as mean ± S.D. by two‐tailed unpaired Student's *t* tests. *P* < 0.05 was considered statistically significant. Significance levels are as follow: *P < 0.05; **P < 0.01; and ***P < 0.001; N.S., not significant.

## Conflict of Interest

The authors declare no conflict of interest.

## Supporting information

Supporting Information

## Data Availability

The data that support the findings of this study are available from the corresponding author upon reasonable request.
