# Supplementary material for: Cilia‐Mediated Insulin/Akt and ST2/JNK Signaling Pathways Regulate the Recovery of Muscle Injury
Source: Adv Sci (Weinh). 2022 Nov 14;10(1):2202632. doi: 10.1002/advs.202202632 (PMC9811445; doi:10.1002/advs.202202632)

ADVANCED SCIENCE
Open Access

Supporting Information

for *Adv. Sci.*, DOI 10.1002/advs.202202632

Cilia-Mediated Insulin/Akt and ST2/JNK Signaling Pathways Regulate the Recovery of Muscle Injury

Daishi Yamakawa, Junya Tsuboi, Kousuke Kasahara, Chise Matsuda, Yuhei Nishimura, Tatsuya Kodama, Naoyuki Katayama, Masatoshi Watanabe and Masaki Inagaki**

Supporting Information

Cilia-mediated Insulin/Akt and ST2/JNK Signaling Pathways Regulate the Recovery of Muscle Injury

Daishi Yamakawa, Junya Tsuboi, Kousuke Kasahara, Chise Matsuda, Yuhei Nishimura, Tatsuya Kodama, Naoyuki Katayama, Masatoshi Watanabe, and Masaki Inagaki**

Figure S1 | Effect of TCHP deficiency on regenerative responses to muscle injury

Representative images of TA muscle cross-sections in WT and *Tchp*$^{-/-}$ mice at the indicated time points following injury by glycerol injection. (**A**) Hematoxylin and eosin staining. Scale bars, 100 µm. (**B**) Immunofluorescence for perilipin (green) and nuclei (Hoechst33342, blue). Scale bar, 100 µm. (**C**) Immunofluorescence for laminin (green), Ki67 (red) and nuclei (Hoechst33342, blue). Scale bars, 50 µm. (**D**) Average cross-section area of myofibers in WT (n = 3) or *Tchp*$^{-/-}$ (n = 5) mice at 0 dpi. Data are the mean ± S.D. N.S., not significant; two-tailed unpaired Student's *t*-tests. (**E**) Immunofluorescence for cilia (Arl13b, green), FAPs (PDGFRα, red) and nuclei (Hoechst33342, blue). Scale bars, 20 µm.

Figure S2 | Effect of TCHP deficiency on adipogenesis or fibrogenesis after muscle injury

(**A**) Representative immunofluorescence images for perilipin (green), PDGFRα (red) and nuclei (Hoechst33342, blue) in TA muscle cross sections of WT or *Tchp*$^{-/-}$ mice following injury with intramuscular injection of cardiotoxin. Scale bar, 100 µm. (**B**) Quantification of the percentages of perilipin-stained area at 21 dpi (WT, n = 3; *Tchp*$^{-/-}$, n =3) in **A**. (**C**) Representative immunofluorescence images for collagen I (green), PDGFRα (red) and nuclei (Hoechst33342, blue) in TA muscle cross sections of WT or *Tchp*$^{-/-}$ mice following injury with intramuscular injection of glycerol. Scale bars, 50 µm. (**D**) Quantification of the percentages of collagen I–stained area at 0, 7 and 21 dpi (WT, n = 3; *Tchp*$^{-/-}$, n =3) in **C**. All data are the mean ± S.D. *p < 0.05, N.S., not significant; two-tailed unpaired Student's *t*-tests.

Figure S3 | Subcellular distributions of flotillin-2 and GM3 in FAPs

PDGFRα$^+$ FAPs were isolated from hindlimbs of WT or *Tchp*$^{-/-}$ mice and cultured *in vitro*, and adipogenic differentiation was induced for 0 or 10 min. (**A**) Representative immunofluorescence images for Sca1 (green), PDGFRα (red) and nuclei (Hoechst33342, blue) of isolated primary FAPs before adipogenic induction. Scale bars, 20 µm. (**B**, **C**) Quantification of the percentage of Sca1$^+$ (**B**) or PDGFRα$^+$ (**C**) cells. Data are the mean ± S.D. from three mice (n > 100 cells each). (**D, F**) Immunofluorescence for flotillin-2 (**D**, green) or GM3 (**F**, green), cilia (Arl13b, red) and nuclei (Hoechst33342, blue). Scale bars, 20 µm. (**E, G**) Quantification of the association of flotillin-2 (**E**) or GM3 (**G**) with the ciliary base labeled with acetylated-tubulin at 0 (white) or 10 min (black) after adipogenic induction. Data are the mean ± S.D. from three mice (n > 50 cells each). **$p < 0.01$, ***$p < 0.001$, N.S., not significant; two-tailed unpaired Student's *t*-tests.

Figure S4 | Effect of Akt kinase inhibition on FAP adipogenesis

PDGFRα$^+$ FAPs isolated from hindlimbs of WT mice were pre-treated with the indicated concentration of MK-2206 or ARQ-092 for 30 min before adipogenic induction. (**A**, **B**) Immunoblotting analysis of whole cell lysates from MK-2206- (**A**) or ARQ-092-treated (**B**) FAPs stimulated with or without adipogenic stimuli for 10 min. (**C, D**) Immunofluorescence for adipocytes (BODIPY, green) and nuclei (Hoechst33342, blue) in FAPs at 5 days after adipogenic induction (**C**) and the percentages of BODIPY$^+$ cells (**D**, n > 100 cells each). Scale bars, 20 μm. Data are the mean ± S.D. from three independent replicates. ***p < 0.001; two-tailed unpaired Student's *t*-tests.

Figure S5 | Effect of TCHP deficiency on FAP fibrogenesis

PDGFRα^{+} FAPs isolated from hindlimbs of WT or *Tchp*$^{-/-}$ mice were cultivated with TGF-β1 for 3 days to induce fibrogenic differentiation or with PBS as a control. (**A–C**) qRT-PCR analysis of RNA isolated from WT or *Tchp*$^{-/-}$ FAPs for *Col1a1* (**A**), *Ccn2* (**B**) or *Tchp* (**C**). White and black columns indicate WT and *Tchp*$^{-/-}$-derived FAPs, respectively. (**D**) Representative immunofluorescence images for collagen I (green) and nuclei (Hoechst33342, blue) of WT or *Tchp*$^{-/-}$ FAPs stimulated with PBS or TGF-β1 for 3 days. Scale bars, 20 μm. (**E**) Percentages of collagen I^{+} cells in **D**. All data are the mean ± S.D. from 3–5 mice. *$p < 0.05$, **$p < 0.01$, ***$p < 0.001$, N.S., not significant; two-tailed unpaired Student's *t*-tests.

Figure S6 | IL13 expression in PDGFRα^{+} FAPs

(**A, B**) Immunofluorescence images of IL4 (red), IL13 (red), IL33 (red), CD45 (green) and nuclei (Hoechst33342, blue) in TA muscle cross sections of WT and *Tchp*$^{-/-}$ mice at 3 dpi. The higher magnifications of the dashed area for the IL13 images are shown in Figure 3E and those of the IL4 and IL33 images are shown in **B**. Scale bars, 200 μm (**A**) and 20 μm (**B**). (**C**) PDGFRα^{+} FAPs isolated from hindlimbs of WT and *Tchp*$^{-/-}$ mice were pretreated with DMSO, 1 μM MK-2206 or 20 μM GANT61 for 30 min and induced for adipogenic differentiation for 5 days. Representative immunofluorescence images for PDGFRα (green), IL13 (red) and nuclei (Hoechst33342, blue) are shown. Scale bars, 20 μm. (**D**) Immunoblotting analysis showed that 20 μM GANT61 treatment suppresses Gli1 expression in FAPs.

Figure S7 | Effect of TCHP deficiency on Treg accumulation

(**A**) Immunofluorescence for FOXP3 (green), CD45 (red) and nuclei (Hoechst33342, blue) at 3 dpi following injury by injection with glycerol (left) or cardiotoxin (right) in TA muscle of WT and *Tchp*$^{-/-}$ mice. Arrowheads indicate FOXP3^{+}CD45^{+} Treg. Scale bars: 100 μm. (**B, C**) Quantification of the percentage of FOXP3^{+}CD45^{+} Tregs in CD45^{+} hematopoietic cells at 3 dpi following injury with glycerol (**B**) or cardiotoxin (**C**) in the TA muscle of WT and *Tchp*$^{-/-}$ mice ($n > 100$ cells each). All data are the mean ± S.D. from three mice. N.S., not significant; two-tailed unpaired Student's *t*-tests.

Figure S8 | Effect of TCHP deficiency on macrophage polarization

F4/80$^+$ monocytes isolated from WT or *Tchp*$^{-/-}$ mice were cultivated with M-CSF for 5 days and then treated with PBS for preparation of M0 macrophages, IFN-γ+LPS for M1 polarization (TNFα$^+$, IL6$^+$) or IL4+IL13 for M2 polarization (Mrc1$^+$, Arg1$^+$) for 2 days. qRT-PCR analysis was performed on RNA isolated from these cells for *Tchp* (**A**), *TNF* (**B**), *Il6* (**C**), *Mrc1* (**D**) and *Arg1* (**E**). All data are the mean ± S.D. from three different mice. **$p < 0.01$, ***$p < 0.001$, N.S., not significant; two-tailed unpaired Student's *t*-tests.

Figure S9 | Effect of IL13 on FAP proliferation after muscle injury

(**A**) Schematic schedule for evaluating the effect of IL13 on the regenerative responses to muscle injury by glycerol injection in the TA muscle of WT mice. (**B, C**) Immunofluorescence for PDGFRα (red) and nuclei (Hoechst33342, blue) at 3 and 7 dpi (**B**) and quantification of the PDGFRα-stained area per 40× view (n = 6 fields each from three mice) (**C**). Scale bar 40 µm. (**D**) Quantification of the number of PDGFRα^{+} FAPs isolated from the hindlimbs of WT mice following culture with PBS or IL13 for 3 or 5 days *in vitro* (n > 200 cells from three independent replicates). All data are the mean ± S.D. N.S., not significant; two-tailed unpaired Student's *t*-tests.

Figure S10 | TCHP-depleted satellite cells or myoblasts

(**A**) Immunofluorescence for desmin (green), PAX7 (red), Ki67 (white) and nuclei (Hoechst33342, blue) in single fibers isolated from the extensor digitorum longus (EDL) muscle of WT and *Tchp*$^{-/-}$ mice, which were cultured in growth medium for 0 or 3 days. Scale bars, 20 µm. (**B–E**) Immunofluorescence for PAX7 (white) and Ki67 (**B**, magenta) or phospho-Histone H3 (Ser10) (**D**, magenta) of primary myoblasts isolated from hindlimbs in wild-type (WT) and *Tchp*$^{-/-}$ mice, and the percentages of PAX7^{+}Ki67^{+} (**D**) or PAX7^{+}phospho-Histone H3 (Ser10)$^{+}$ myoblasts (**E**) (n > 50 cells each). Scale bars, 20 µm. (**F**) Quantification of myoblasts isolated from WT and *Tchp*$^{-/-}$ mice following culture for 3 (white) or 7 days (black) in growth medium (n = 5 fields each). (**G**) Phase contrast images of myoblasts isolated from WT and *Tchp*$^{-/-}$ mice following culture in myogenic differentiation medium for 0 or 2 days. Scale bar, 100 µm. (**H**) Immunofluorescence for desmin (green), vimentin (red) and nuclei (Hoechst33342, blue) of myoblasts isolated from WT and *Tchp*$^{-/-}$ mice following culture in myogenic differentiation medium for 0 or 2 days. Scale bar, 20 µm. (**I**) The percentage of desmin^{+} myoblasts analyzed in **H**. White and black bars indicate WT and *Tchp*$^{-/-}$-derived myoblasts, respectively (n > 100 cells each). All data are the mean ± S.D. from three mice. N.S., not significant; two-tailed unpaired Student's *t*-tests.

Figure S11 | Effect of IL13 on adipogenesis or fibrogenesis after muscle injury

(**A**) Schematic of the schedule for evaluating the effect of IL13 on the regenerative responses to muscle injury by glycerol injection in the TA muscle of WT mice. (**B**) Immunofluorescence for perilipin (green) and nuclei (Hoechst33342, blue) in the TA muscle of PBS- or IL13-injected mice at 3 and 7 dpi. Scale bars: 40 μm. (**C**) Quantification of the perilipin-stained area per 40× view in **B** (n = 6 fields each). (**D**) Representative immunofluorescence for collagen I (green), PDGFRα (red) and nuclei (Hoechst33342, blue) in the TA muscle of PBS- or IL13-injected mice at 3 and 7 dpi. Scale bars: 20 μm. (**E**) Quantification of the collagen I–stained area in **D** (n = 6 fields each). All data are the mean ± S.D. from three mice. *p < 0.05, N.S., not significant; two-tailed unpaired Student's *t*-tests.

Figure S12 | Effect of IL13 on adipogenesis or fibrogenesis in FAPs *in vitro*

(**A**) PDGFRα$^+$ FAPs isolated from hindlimbs of WT mice were treated with TGF-β1 for 3 days to induce fibrogenic differentiation in the presence of PBS or IL13. Representative immunofluorescence images for collagen I (green) and nuclei (Hoechst33342, blue) are shown. Scale bars, 20 µm. (**B**) Percentage of collagen I$^+$ cells in **A.** (**C**) qRT-PCR analysis of *Ccn2* in RNA isolated from FAPs in **A**. (**D**) PDGFRα$^+$ FAPs isolated from hindlimb of WT mice were induced for adipogenic differentiation for 5 days in the presence of PBS or IL13. Representative immunofluorescence images for BODIPY (green) and nuclei (Hoechst33342, blue) are shown. Scale bars, 20 µm. (**E**) Percentages of the BODIPY$^+$ cells in **D** ($n > 200$ cells each). All data are the mean ± S.D. from three different mice. *$p < 0.05$, ***$p < 0.001$, N.S., not significant; two-tailed unpaired Student's *t*-tests.

Table S1. Sequences of primers used for quantitative real-time PCR.

Name	Sequence (5′–3′)	Source
Mouse Amphiregulin Fwd	TGG CAT CGG CAT CGT TAT CA	This paper
Mouse Amphiregulin Rev	GCA TTG TCC TCA GCT AGG CA	This paper
Mouse Arg1 Fwd	CTC CAA GCC AAA GTC CTT AGA G	Toda et al. 2021
Mouse Arg1 Rev	GGA GCT GTC ATT AGG GAC ATC A	Toda et al. 2021
Mouse CTGF Fwd	AGC AGC TGG GAG AAC TGT GT	This paper
Mouse CTGF Rev	GCT GCT TTG GAA GGA CTC AC	This paper
Mouse Col1a1 Fwd	GAG CGG AGA GTA CTG GAT CG	This paper
Mouse Col1a1 Rev	GTT CGG GCT GAT GTA CCA GT	This paper
Mouse Desmin Fwd	CGA GCT CTA CGA GGA GGA GA	This paper
Mouse Desmin Rev	GCC TCT GCA GGT CGT CTA TC	This paper
Mouse Follistatin Fwd	AAA ACC TAC CGC AAC GAA TG	This paper
Mouse Follistatin Rev	CTA GTT CCG GCT GCT CTT TG	This paper
Mouse GAPDH Fwd	AAC TTT GGC ATT GTG GAA GG	Yamakawa et al. 2021
Mouse GAPDH Rev	GGA TGC AGG GAT GAT GTT CT	Yamakawa et al. 2021
Mouse Gli1 Fwd	TGT GTG AGC AAG AAG GTT GC	This paper
Mouse Gli1 Rev	ATG GCT TCT CAT TGG AGT GG	This paper
Mouse IL4 Fwd	CCA TAT CCA CGG ATG CGA CA	This paper
Mouse IL4 Rev	AGG ACG TTT GGC ACA TCC AT	This paper
Mouse IL6 Fwd	CCG GAG AGG AGA CTT CAC AG	This paper
Mouse IL6 Rev	CAG AAT TGC CAT TGC ACA AC	This paper
Mouse IL8 Fwd	GGA TCC TGA TGC TCC ATG GG	This paper
Mouse IL8 Rev	CAG AAG CTT CAT TGC CGG TG	This paper
Mouse IL10 Fwd	CAG AGA AGC ATG GCC CAG AA	This paper
Mouse IL10 Rev	CTT CAG CTT CTC ACC CAG GG	This paper
Mouse IL13 Fwd	CAC ACA AGA CCA GAC TCC CC	This paper
Mouse IL13 Rev	GTT GGT CAG GGA ATC CAG GG	This paper
Mouse IL15 Fwd	GCC TCT TCA TGG TCC TTG CT	This paper
Mouse IL15 Rev	GAT GAG CTG GCT ATG GCG AT	This paper
Mouse IL33 Fwd	TTC TCT GCC TAT CCA CGG GA	This paper
Mouse IL33 Rev	TAC TCA GGG AGG CAG GAG AC	This paper
Mouse Mrc1 Fwd	TCG GTG GAC TGT GGA CGA GCA	Jia et al. 2013
Mouse Mrc1 Rev	TCC CGC CTT TCG TCC TGG CA	Jia et al. 2013
Mouse Pax7 Fwd	GAC TCC GGA TGT GGA GAA AA	This paper
Mouse Pax7 Rev	GAG CAC TCG GCT AAT CGA AC	This paper
Mouse Ptch1 Fwd	CAG GAC AAC CTC AGC TGT CA	This paper
Mouse Ptch1 Rev	TGA GAA CTG GGA GAG CAG GT	This paper
Mouse Tchp Fwd	AAG TCA GCC CGA GAA GAA CA	This paper
Mouse Tchp Rev	TCA CGT GCT TCT TGT GAA GG	This paper
Mouse TNF-α Fwd	CCC CAA AGG GAT GAG AAG TT	This paper
Mouse TNF-α Rev	GGT CTG GGC CAT AGA ACT GA	This paper
Mouse Wnt 5a Fwd	CGC TTC GCT TGA ATT CCT CG	This paper
Mouse Wnt 5a Rev	AAT GGG CTT CTT CAT GGC GA	This paper

Figure S1.

Yamakawa et al.

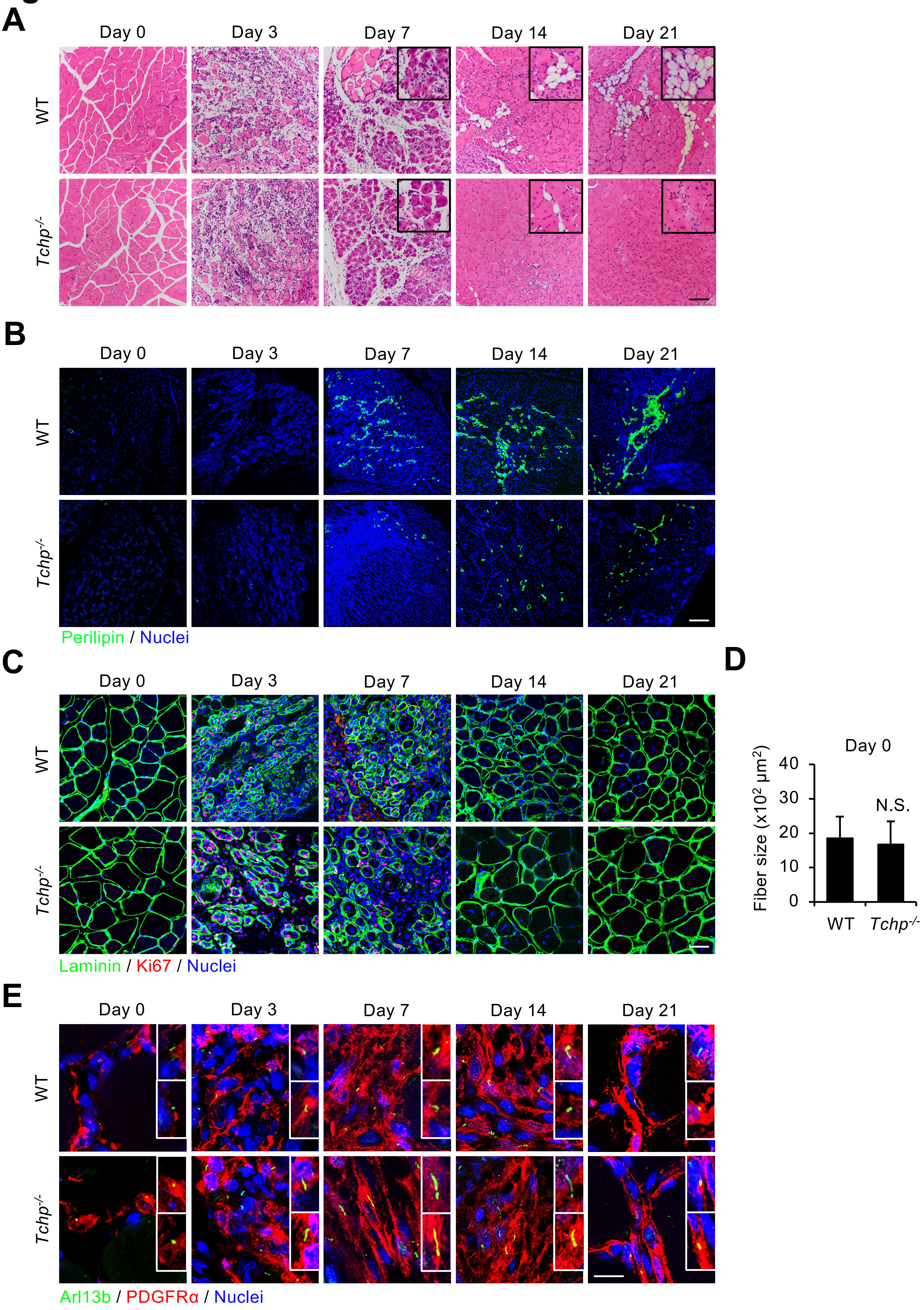

Figure S2. **Yamakawa et al.**

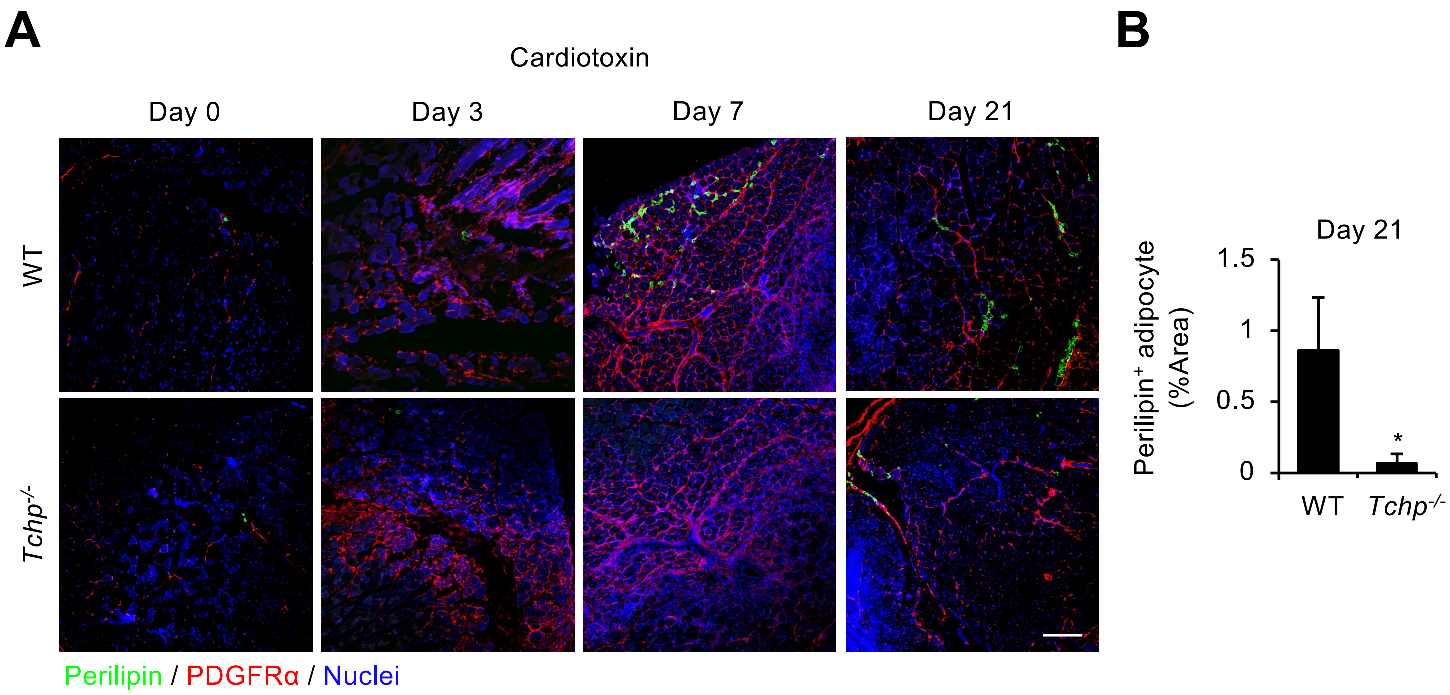

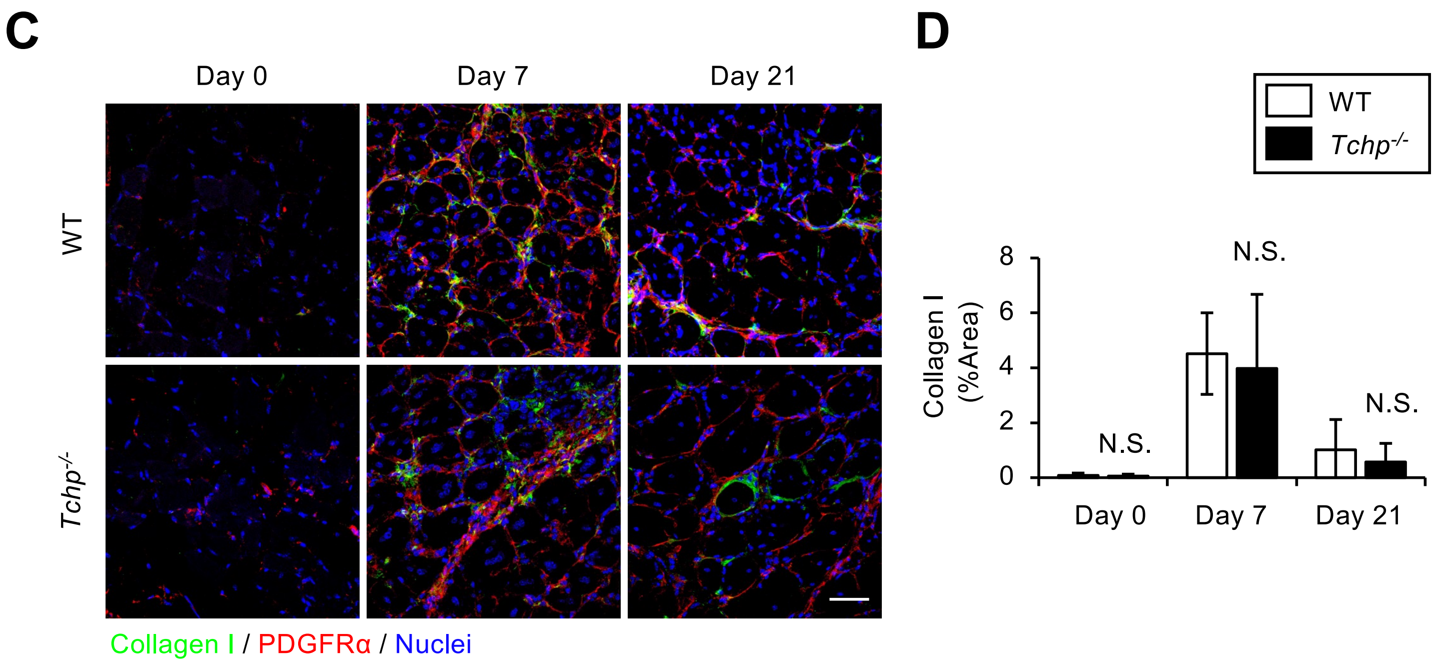

Figure S3.

Yamakawa et al.

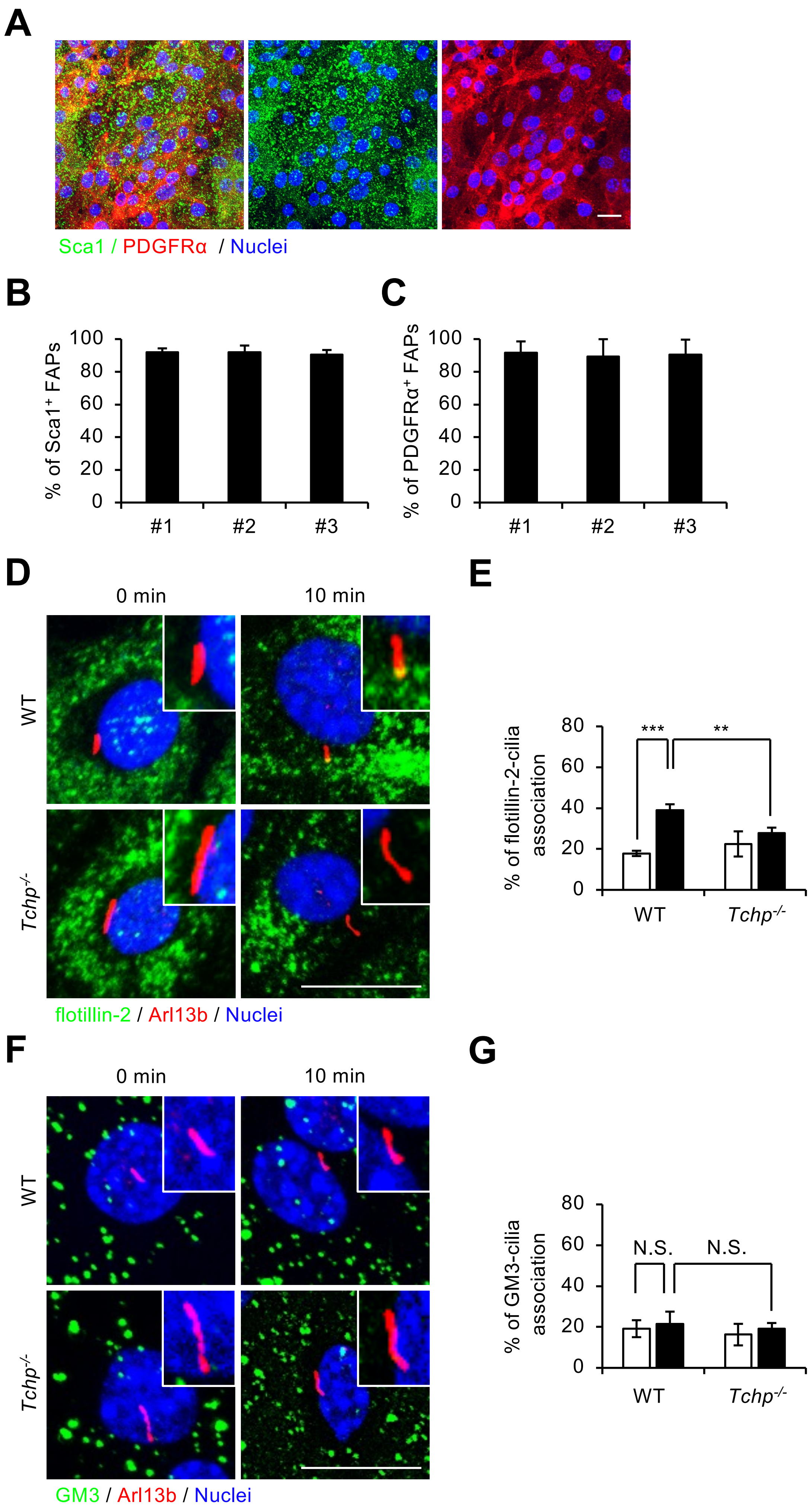

Figure S4.

Yamakawa et al.

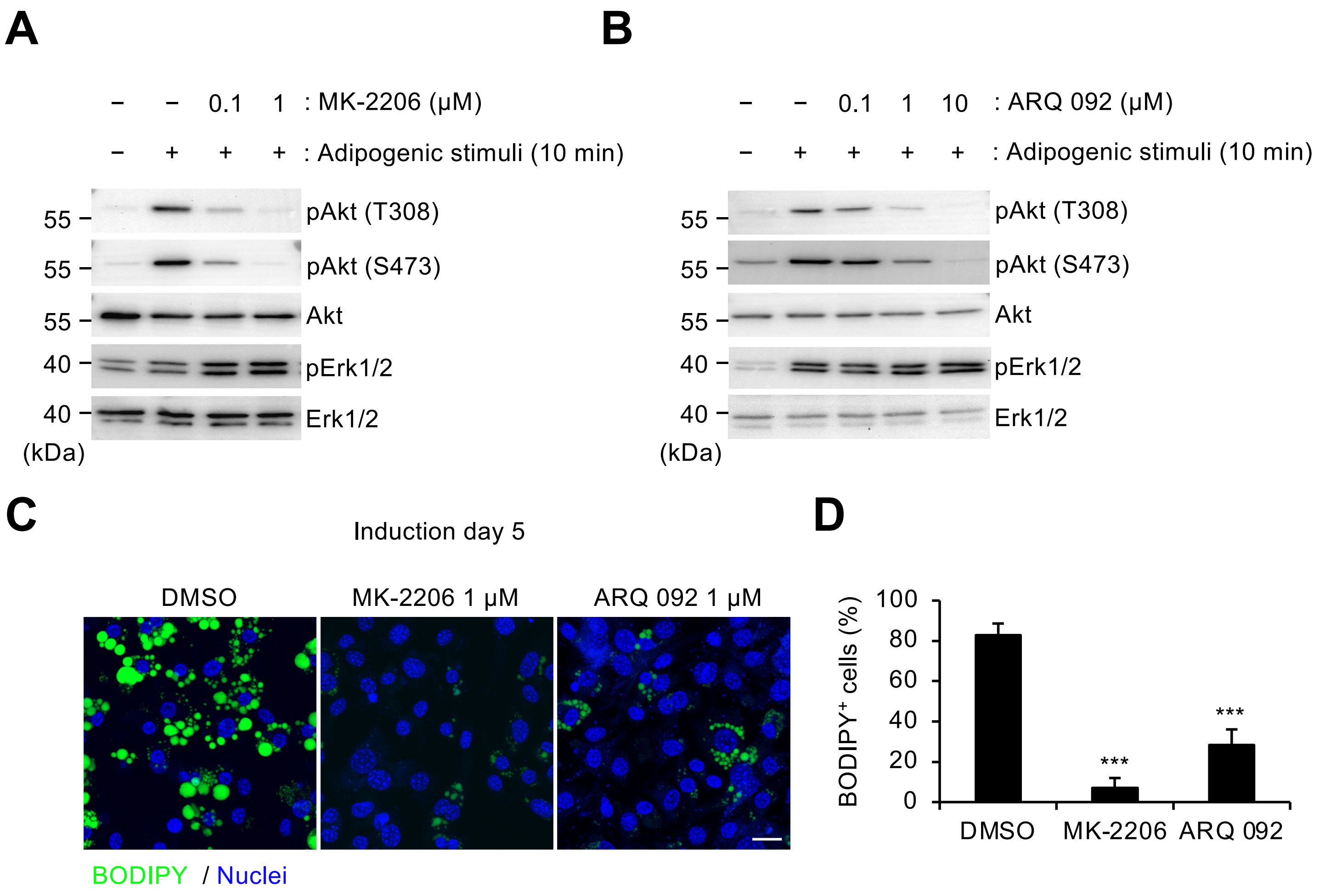

Figure S5. Yamakawa et al.

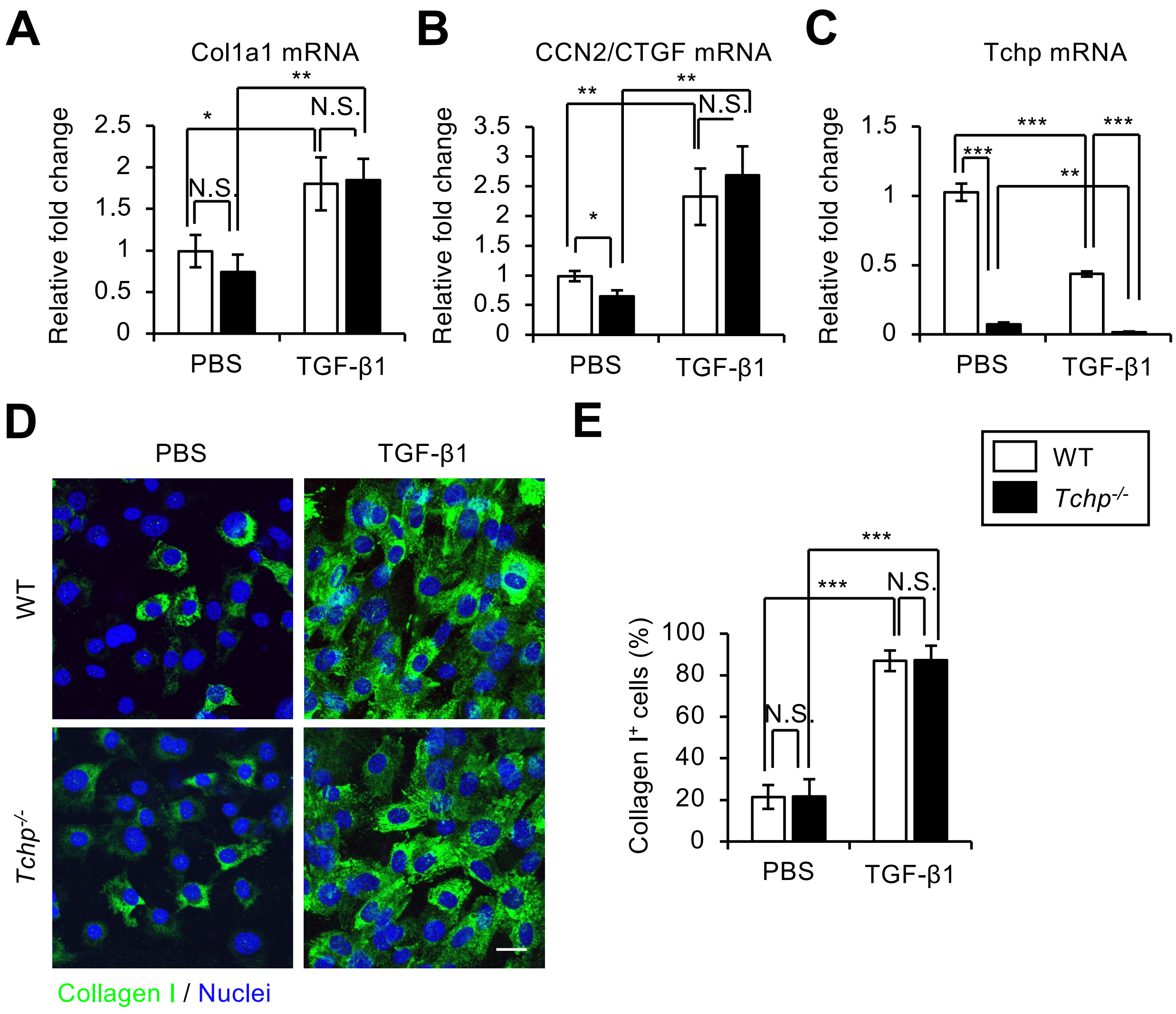

Figure S6.

Yamakawa et al.

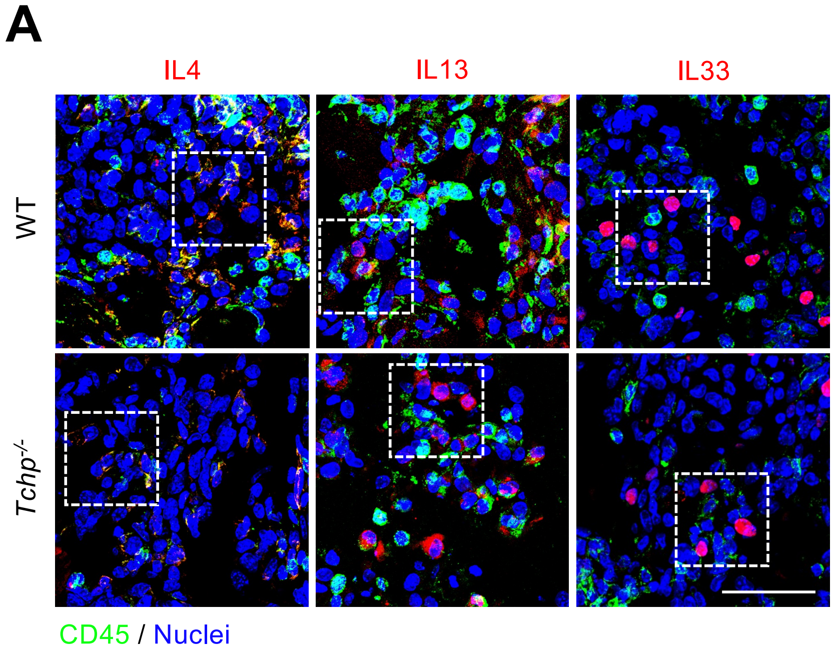

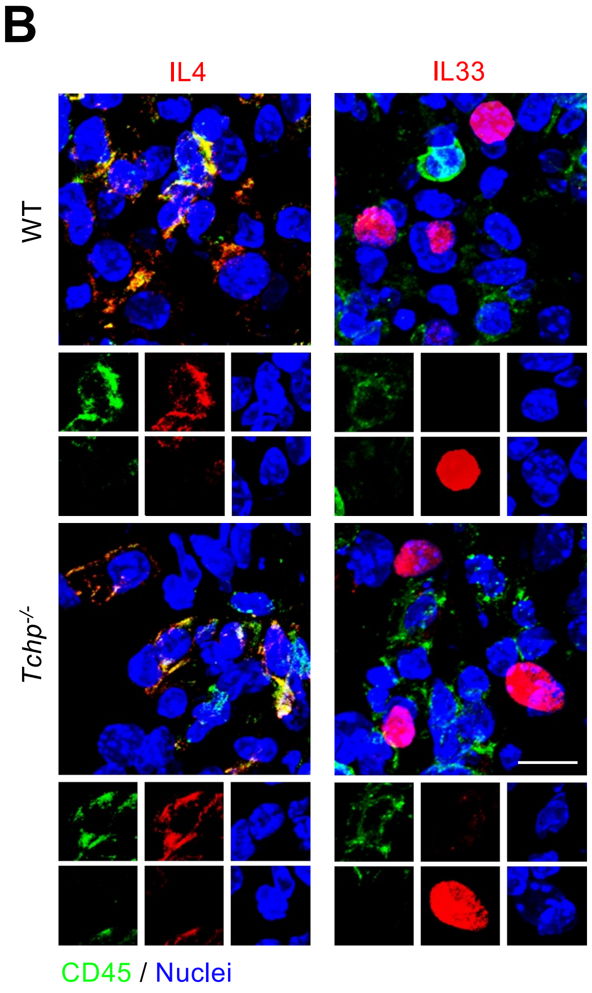

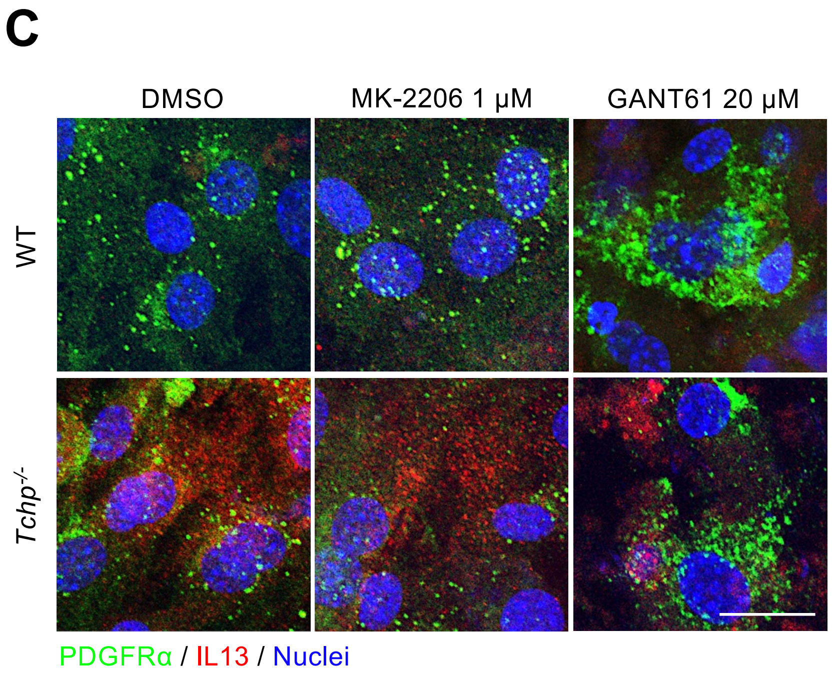

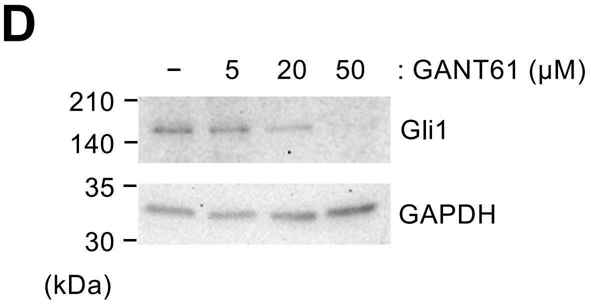

Figure S7. **Yamakawa et al.**

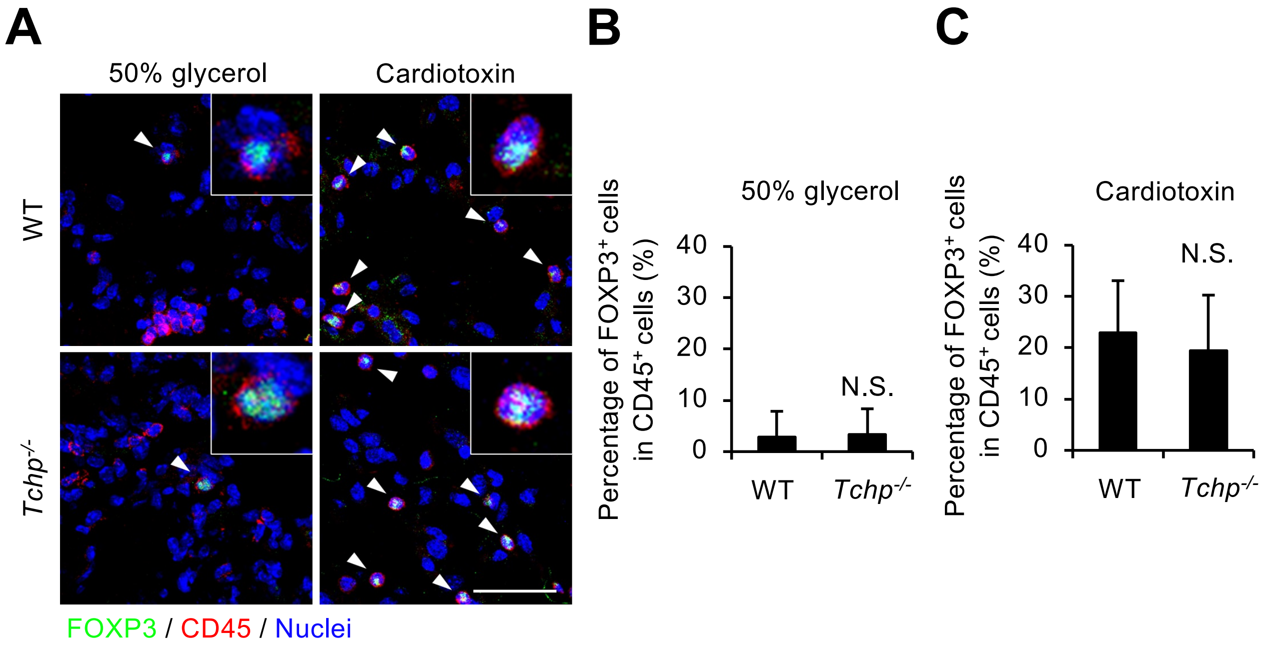

Figure S8. Yamakawa et al.

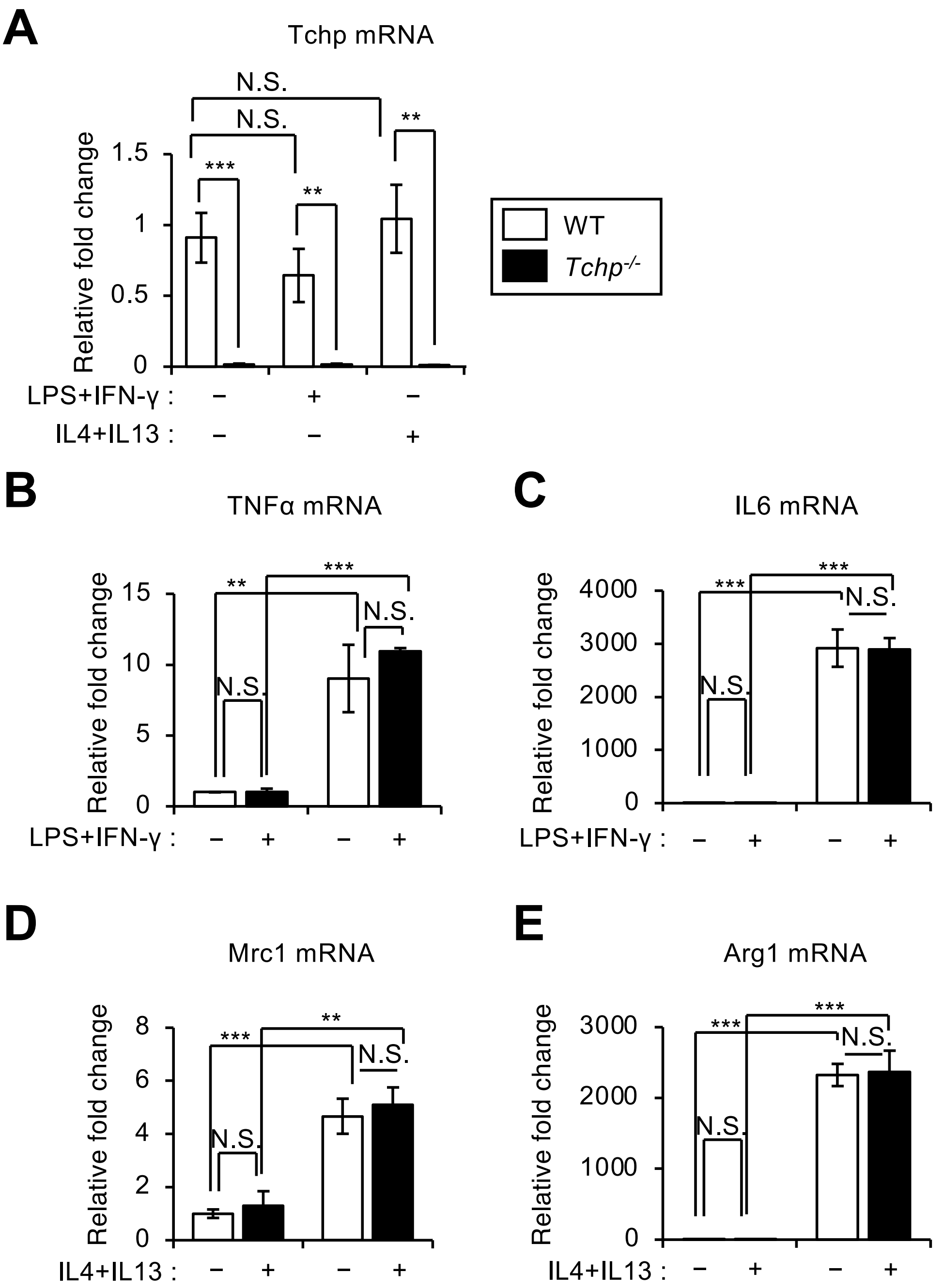

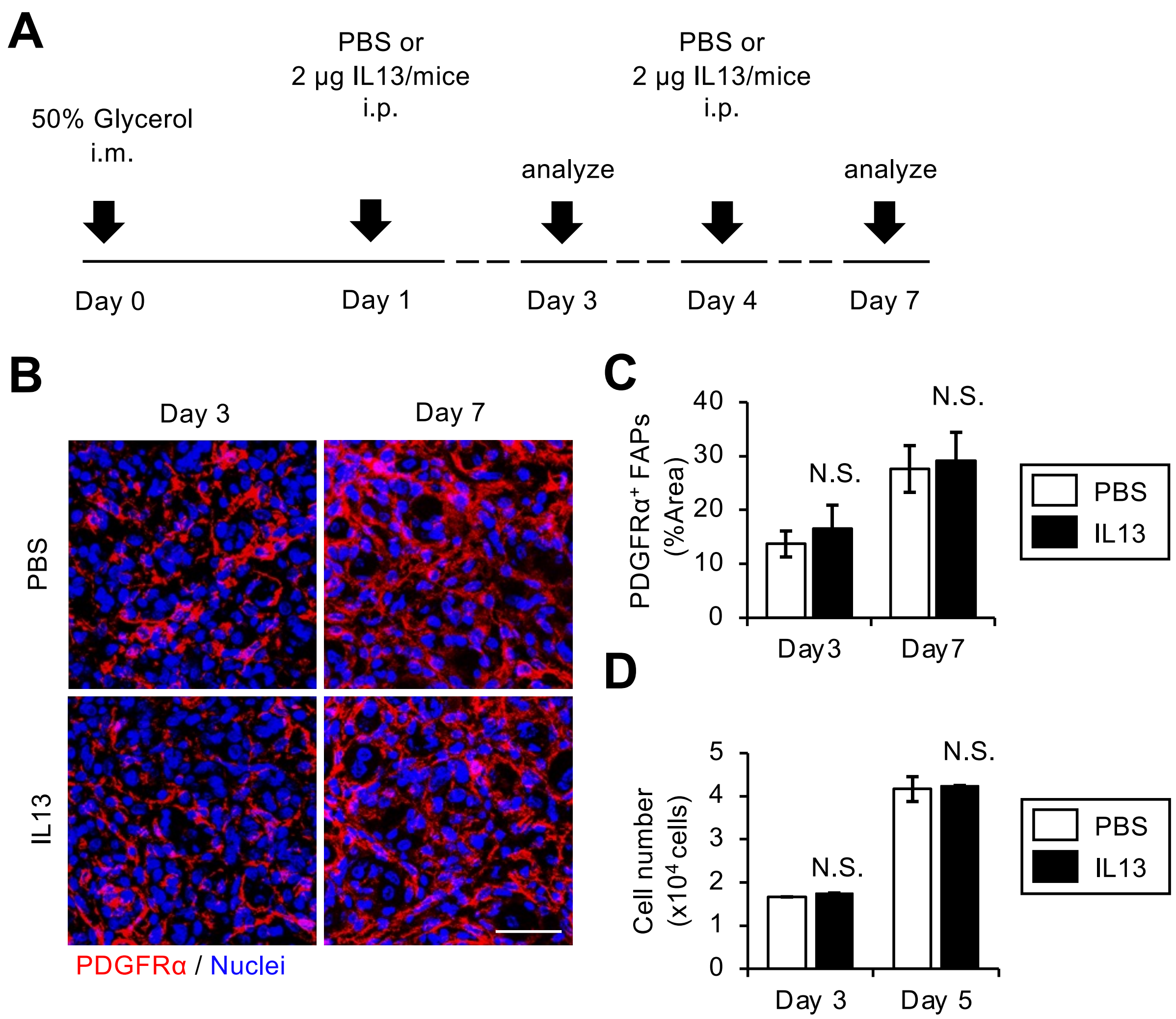
A
50% Glycerol
i.m.
PBS or
2 μg IL13/mice
i.p.
analyze
PBS or
2 μg IL13/mice
i.p.
analyze
Day 0
Day 1
Day 3
Day 4
Day 7
B
Day 3
Day 7
PBS
IL13
PDGFRα / Nuclei
C
PDGFRα+ FAPs (%Area)
40
30
20
10
0
N.S.
N.S.
Day3
Day7
PBS
IL13
D
Cell number (x10⁴ cells)
5
4
3
2
1
0
N.S.
N.S.
Day 3
Day 5
PBS
IL13

Figure S10. Yamakawa et al.

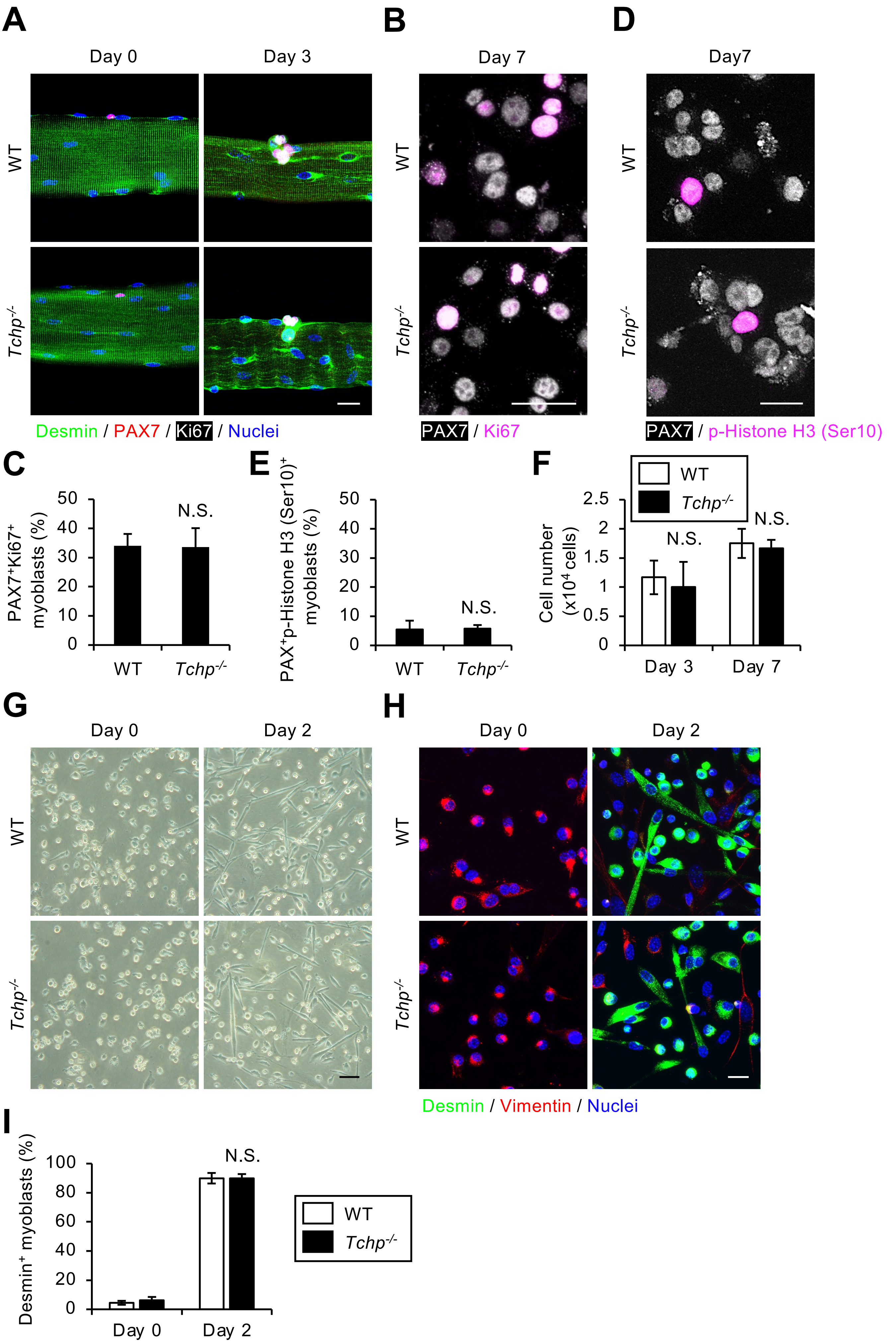

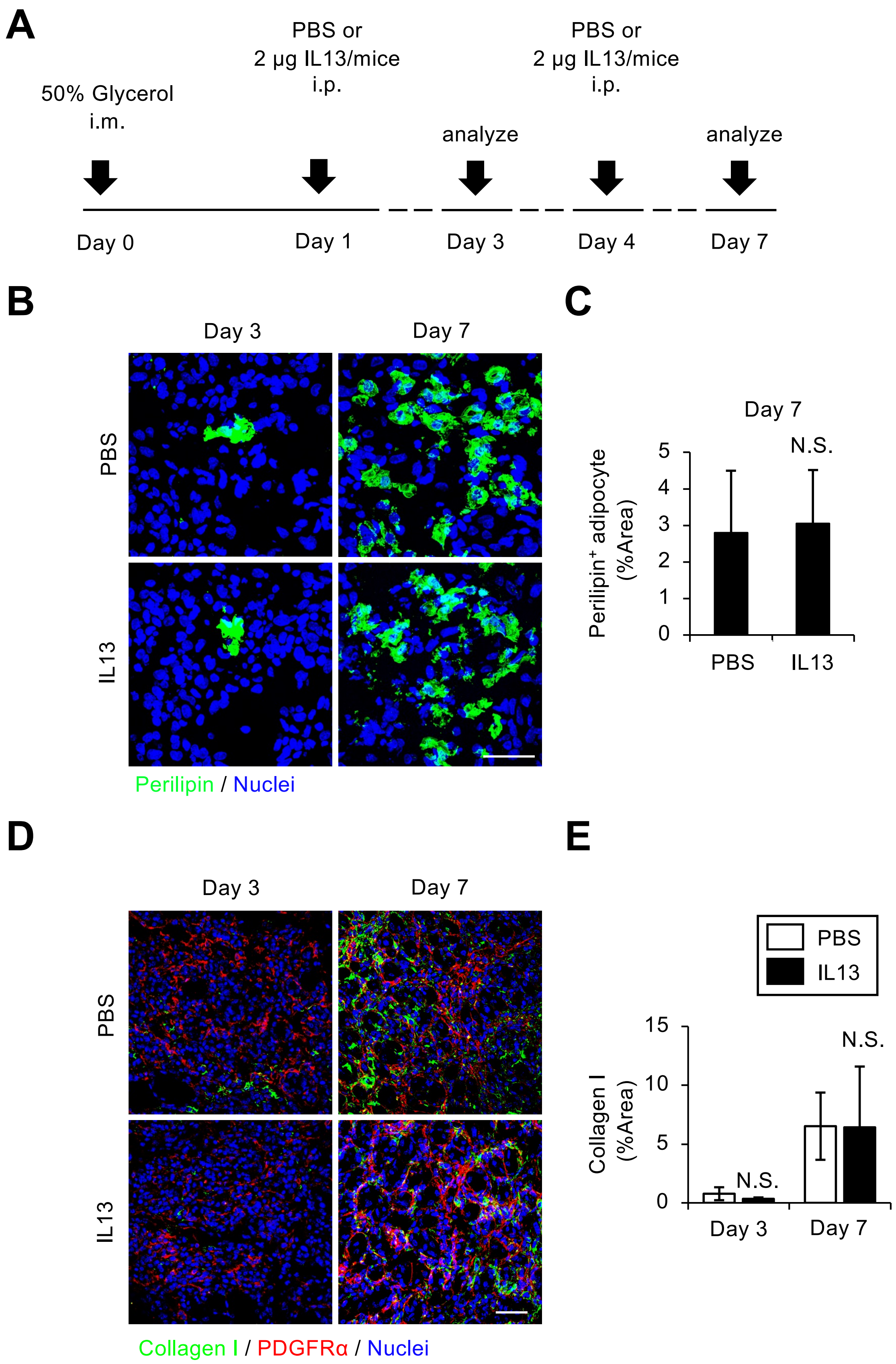
A
PBS or
2 µg IL13/mice
i.p.
50% Glycerol
i.m.
analyze
PBS or
2 µg IL13/mice
i.p.
analyze
Day 0
Day 1
Day 3
Day 4
Day 7
B
Day 3
Day 7
PBS
IL13
Perilipin / Nuclei
C
Day 7
N.S.
Perilipin+ adipocyte (%Area)
5
4
3
2
1
0
PBS
IL13
D
Day 3
Day 7
PBS
IL13
Collagen I / PDGFRα / Nuclei
E
PBS
IL13
15
10
5
0
Collagen I (%Area)
N.S.
N.S.
Day 3
Day 7

Figure S12. Yamakawa et al.

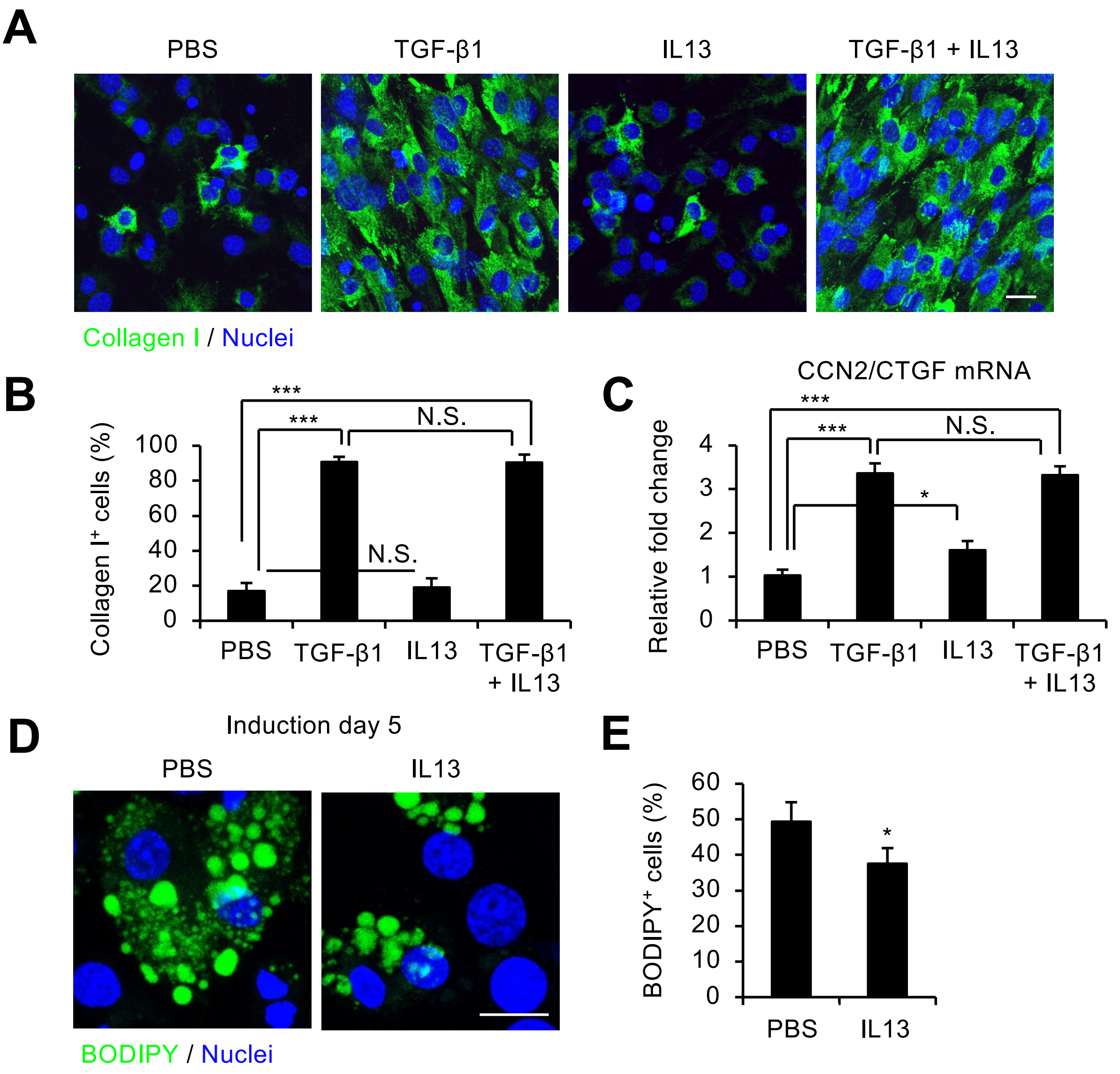

Supplement: Supplementary file 1 — Supporting Information [file ADVS-10-2202632-s001.pdf]
